# Hypoxic stimulation of *DCLK1* transcription and alternative-promoter switching fuels tumor malignancy in clear cell renal cell carcinoma

**DOI:** 10.1038/s41419-025-07916-2

**Published:** 2025-08-07

**Authors:** Jiannan Yao, Xuying Huang, Qianqian Sun, Wenjing Zhao, Nathaniel Weygant, Xiaona Fan, Heshu Liu, Zeru Xiao, Rui Yan, Yang Ge, Guangyu An, Jian Liu

**Affiliations:** 1 Department of Oncology, Beijing Chao-Yang Hospital, Capital Medical University, 100020, Beijing, China; 2 Medical Research Center, Beijing Institute of Respiratory Medicine and Beijing Chao-Yang Hospital, Capital Medical University, 100020, Beijing, China; 3 Fujian University of Traditional Chinese Medicine, Academy of Integrative Medicine, 350122, Fuzhou, Fujian China; 4 Fujian Key Laboratory of Integrative Medicine in Geriatrics, 350122, Fuzhou, Fujian China

**Keywords:** Cancer, Cell signalling

## Abstract

Alterations in the diversity and abundance of oncogenic gene transcripts are key factors driving tumor initiation and progression. DCLK1, an emerging cancer stem cell marker, is activated during tumorigenesis, triggering cancer stemness and metastasis. It produces long or short isoforms through selective usage of alternative promoters (α- or β-promoter). However, the mechanism mediating DCLK1 activation and choice of AP model in cancer remains unclear. Herein, we reveal that DCLK1 is significantly activated, with a concomitant alternative-promoter model switch (β-to-α) towards the long variants (isoform 1 and 2) in clear cell renal cell carcinoma (ccRCC). During tumorigenesis, the α-promoter is initiated with an improved probability from 31.6% to 61.1%, whereas the initiation probability of the β-promoter declined from 68.4% to 38.9%. Mechanistically, this alteration is mediated by a hypoxia-HIF2α-PLOD2 axis, which further activates β-catenin to selectively bind and activate the α-promoter. Our findings also showed that the hyperactivated PLOD2-DCLK1-L axis in ccRCC is correlated with a higher EMT signature and predicts an unfavorable prognosis in ccRCC patients, while disrupting this signaling by pharmacological targeting of DCLK1-L significantly attenuated cancer malignancy both in vitro and in vivo. These findings couple hypoxia signaling to oncogenic alternative switching and highlight DCLK1-L as a promising therapeutic target for hypoxic PLOD2-rich ccRCCs.

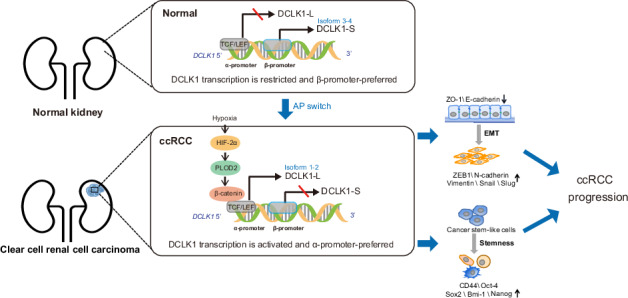

## Introduction

Renal cell carcinoma (RCC) is among the most common genitourinary cancers in adults, causing 179,368 deaths worldwide in 2020, with the majority of deaths due to metastatic spread [1]. Clear cell RCC (ccRCC) is the most frequent histological subtype, representing 70–90% of total cases and accounting for 83%-88% of all metastatic cases [2, 3]. The vast majority of ccRCCs are associated with functional inactivation of the von Hippel-Lindau (VHL) tumor suppressor gene and deregulation of hypoxia signaling [4].

For localized ccRCC, partial or radical nephrectomy is the primary treatment modality. Nevertheless, approximately 30% of patients are detected with metastases at diagnosis, and up to 40% of post-surgery cases experience recurrence or metastasis [5]. Given the chemo- and radio-resistance of ccRCC, therapeutic options for metastatic ccRCC are limited, with a 5-year overall survival (OS) rate as low as 12% [6]. Current standard care for metastatic ccRCC primarily involves tyrosine receptor kinase inhibitor drugs (VEGF-TKI) targeting vascular endothelial growth factor (VEGF) receptors, such as sunitinib and sorafenib [7, 8]. However, 30% of patients exhibit innate resistance to VEGF-TKIs, and 70% of initially responsive patients eventually develop resistance [9]. Thus, more effective therapeutic strategies are urgently needed for metastatic ccRCC.

Doublecortin-like kinase 1 (DCLK1) is a promising therapeutic target for cancer [10]. Initially, DCLK1 was reported to play an essential role in neurogenesis and neuronal migration [11–13], and was subsequently identified as a cancer stem cell (CSC) marker in gastrointestinal cancers [14]. To date, widespread DCLK1 upregulation has been reported across various solid malignancies, including colorectal cancer [15–17], breast cancer [18, 19], pancreatic cancer [20, 21], renal cancer [22, 23], as well as hepatocellular carcinoma [24] and esophageal squamous cell carcinoma [25]. Through its role in promoting cancer stemness and EMT properties, DCLK1 overexpression drives tumorigenesis, proliferation, metastasis, and drug resistance, leading to cancer recurrence and poor patient survival [10].

Beyond transcriptional activation, alternative splicing (AS) events, including alternative promoters (AP) and exon skipping (ES), also play essential roles in determining the diversity and abundance of DCLK1 transcripts in cancer [26]. The human DCLK1 gene is transcribed via two alternative promoters: the 5’ (α) promoter or the intron V (β) promoter, generating the longer DCLK1-L or the shorter DCLK1-S pre-mRNAs, respectively. These pre-mRNAs undergo further splicing and are ultimately translated into the DCLK1-L protein variants (isoform 1 or isoform 2, 80–82 kDa) or the DCLK1-S protein variants (isoform 3 or isoform 4, 47–52 kDa). The difference between the two DCLK1-L isoforms originated from a skipped or retained exon, a variation also observed in the two DCLK1-S isoforms [27]. Although derived from the same gene, evidence suggests distinct subcellular distributions and functional differences between DCLK1-S and DCLK1-L isoforms in cancer [10].

Despite consistent transcriptional activation, DCLK1 promoter selection and activation can vary across different cancer types. In human colorectal cancer, hypermethylation of the 5’ (α)-promoter results in loss of DCLK1-L expression and preferential use of the β-promoter, leading to upregulation of DCLK1-S transcripts [28]. Similarly, a recent study revealed significant upregulation of DCLK1-S, but not DCLK1-L, in esophageal squamous cell carcinoma (ESCC) [25]. In contrast, substantial upregulation of DCLK1-L variants has been observed in pancreatic ductal adenocarcinoma [29] and renal cell carcinoma [22, 23, 30]. Despite variability in DCLK1 promoter activation, the underlying mechanism remains largely unexplored. A recent study suggests that β-catenin selectively activates the α-promoter by binding to TCF4/LEF sites, whereas NF-κBp65 specifically binds to the NF-κB cis-element to activate the β-promoter in colon cancer cells [31].

Recent research suggests that DCLK1 may serve as a potential therapeutic target in ccRCC [22, 29, 30]. DCLK1 is prevalently upregulated in RCC, with its expression being more prominent in ccRCC compared to papillary and chromophobic RCCs [22]. Intriguingly, unlike the predominant upregulation of the DCLK1-S isoforms in colorectal cancer and esophageal squamous cell carcinoma, ccRCC shows substantial upregulation of DCLK1-L variants [22, 23], suggesting a potential cancer-specific activation of α-promoter in ccRCC. However, the mechanism governing DCLK1-L activation is still obscure.

In the present study, we revealed that DCLK1 is significantly activated in ccRCC, with an obvious AP model switching towards the α-promoter during tumorigenesis, leading to marked activation of the DCLK1-L isoforms. Furthermore, we disclose that hypoxia signaling, co-activated by microenvironmental hypoxia [32, 33] and pVHL inactivation [34–36] in ccRCC, drives preferential activation of the DCLK1 α-promoter. We further revealed that procollagen lysyl hydroxylase 2 (PLOD2), a well-documented mediator of hypoxia-induced EMT [37, 38], acts downstream of HIF2α and plays an essential role in driving hypoxia-dependent preferential activation of DCLK1-L isoforms in ccRCC. This biased activation relied on β-catenin’s preferential occupancy and transactivation of the DCLK1 α-promoter. We also identified that both DCLK1-L variants exhibit pro-oncogenic roles, promoting cancer stemness and aggressiveness, and that DCLK1-L inhibition can significantly attenuate ccRCC malignancy in vitro and in vivo.

## Materials and Methods

### Cell culture

Human clear cell renal cancer cell lines 786-O, 769-P, and OS-RC-2, along with the human embryonic kidney cell line HEK293T were acquired from the National Collection of Authenticated Cell Cultures (Beijing, China). The 786-O, 769-P and OS-RC-2 cells were maintained in RPMI-1640 medium (SIGMA, Vienna, Austria), and HEK293T was maintained in DMEM medium (Gibco, California, USA). All media were supplemented with 10% fetal bovine serum (FBS; Ausbian, Australia) and 1% penicillin-streptomycin mixture (Solarbio, Beijing, China). The cell lines were incubated at 37 °C in a humidified incubator with 5% CO2. For hypoxia treatment, cells were cultured under 1% O2, 94% N2, and 5% CO2 for 48 hours.

### CRISPR/Cas9-mediated ablation of DCLK1-L

CRISPR/Cas9 technology was employed to specifically ablate DCLK1-L. DCLK1-L specific sgRNA sequences were annealed and cloned into the lentiCRISPRv2 plasmid, which was then co-transfected with packaging plasmids psPAX2 and pMD2G into HEK293T cells using Lipofectamine 3000 (Invitrogen, Carlsbad, CA, USA) to produce lentivirus particles. Approximately 72 hours post-transfection, viral particles were harvested and filtered through a 0.45 μm filter. After 3 days of infection, infected ccRCC cells were screened with 2 μg/ml puromycin (Gibco, USA) followed by confirmation via Western blotting. The DCLK1-L-specific sgRNA sequences were as follows:

DCLK1-L-SgRNA1: Oligo1 5’-CACCGCTTGACTTCACCGACCAGT-3’

Oligo2 3’-CGAACTGCAAGTGGCTGGTCACAAA-5’

DCLK1-L-SgRNA2: Oligo1 5’-CACCGGGTGAAACGCCTGTACACGT-3’

Oligo2 3’-CCCACTTTGCGGACATGTGCACAAA-5’

### Overexpression of DCLK1 isoform 1 and isoform 2

The coding sequences (CDS) of DCLK1 isoform 1 and isoform 2 were cloned into the pCDH-MSCV-MCS-EF1α-puro plasmid to construct lentiviral overexpression vectors, which were subsequently co-transfected with the package plasmids pMD2G and psPAX2 to generate lentiviral particles. After 3 days of infection, transfected cells were screened with 2 μg/ml puromycin to establish stable DCLK1 isoform1 and isoform 2 overexpression cell lines.

### Overexpression and knockout of PLOD2 and β-catenin

The human PLOD2 overexpression lentiviral vector (pLV(Exp)- mCherry/ Neo-EF1A > hPLOD2), the human β-catenin overexpression lentiviral vector (pLV(Exp)-mCherry/Neo-EF1A > hCTNNB1), the human β-catenin sgRNA lentiviral vector (YKO-RP003-hCTNNB1[gRNA 5-6]), and their respective control vectors were purchased from UBIGENE (Guangzhou, China). To achieve genetic ablation of human PLOD2, three pairs of specific sgRNA were designed and cloned into the lentiCRISPRv2 plasmid. All lentivirus particles were produced in HEK293T cells. After 3 days of infection, ccRCC cells infected with lentivirus particles for PLOD2 overexpression, β-catenin overexpression or β-catenin knockout were subsequently selected with 400 μg/mL G418 (Solarbio, Beijing, China), while those infected with PLOD2 sgRNA lentivirus particles were selected with 2 μg/ml puromycin. The PLOD2 sgRNA sequences were as follows:

PLOD2-sgRNA-1: Oligo1 5’-CACCGATATTTCAATTATACTGTGA-3’

Oligo2 3’-CTATAAAGTTAATATGACACTCAAA-5’

PLOD2-sgRNA-2: Oligo1 5’-CACCGGTAGCAACAAAAGAAAGTGA-3’

Oligo2 3’-CCATCGTTGTTTTCTTTCACTCAAA-5’

PLOD2-sgRNA-3: Oligo1 5’-CACCGGTTGTGGCTGAGAAGATGAG-3’

Oligo2 3’- CCAACACCGACTCTTCTACTCCAAA-5’

### Protein extraction and Western blotting

Whole-cell protein extracts were prepared using RIPA lysis buffer (Beijing Solarbio Science & Technology Co., Ltd.) and quantified with a bicinchoninic acid (BCA) assay kit (Thermo Fischer Scientific, Inc.). Next, 40 µg of protein per lane was denatured, loaded onto a 10% gel, separated by SDS-PAGE, and transferred to a PVDF membrane using a semi-dry transfer system (EMD Millipore) at 200 mA for 1.5 hours. Membranes were incubated with primary antibodies at 4˚C overnight, then with horseradish peroxidase-conjugated secondary IgG antibodies (1:8,000; Santa Cruz Biotechnology, Inc.) at room temperature for 1 hour. Protein signals were detected using an enhanced chemiluminescence kit (Applygen Technologies Inc.). Details of all antibodies used are provided in Table S1.

### RNA extraction and Real Time-quantitative PCR

Total RNA was extracted using TRIeasy reagent (Yeasen Biotechnology, Shanghai, China), according to the manufacturer’s instructions. RNA concentration was determined using a Nanodrop 2000 spectrophotometer. RNA samples with OD 260/280 ratios ranging from 1.8 to 2.0 were considered to be of acceptable quality. Reverse transcription was performed using 1 µg of total RNA with the cDNA Synthesis SuperMix (Yeasen Biotechnology, Shanghai, China). Primers were synthesized by Rui Biotech (Beijing, China). RT-qPCR was carried out using qPCR SYBR Green Master Mix (Yeasen Biotechnology, Shanghai, China). Each sample was analyzed in triplicate, and the relative expression levels of RNA between different samples were determined using the 2^−ΔΔCT^ method, with β-actin serving as the internal control. Details of all primers used are listed in Table S2.

### Transwell migration and invasion assay

The migratory and invasive abilities of ccRCC cells were assessed using the Transwell chamber assay (Corning, USA). For the migration assay, serum-free medium containing 3 × 10^4 cells was added to the upper chamber. For the invasion assay, the upper chamber was coated with a 1:8 mixture of reduced growth factor Matrigel and PBS and incubated at 37 °C for 2 hours. After incubation, serum-free medium containing 3 × 10^4 cells was seeded into the upper chamber. The lower chamber was filled with complete medium containing 10% FBS to serve as a chemoattractant. Following 24 hours of incubation at 37 °C, cells that had migrated to the underside were fixed with 4% paraformaldehyde for 15 minutes, then stained with 0.1% crystal violet. Finally, 3–5 fields per well were randomly selected for microscopic counting.

### Human ccRCC cDNA microarray assay

A human ccRCC tissue cDNA microarray (catalog number: cDNA-HKidE030CS01) was acquired from Shanghai Outdo Biotech. This microarray contained cDNA samples from 15 ccRCC cancer patients. The expression levels of DCLK1-L and PLOD2 in these cDNA samples were measured by real-time quantitative PCR.

### Chromatin immunoprecipitation (ChIP) followed by qPCR assay

ChIP experiments were performed using a ChIP-IT® Express kit (Active Motif, Carlsbad, CA, USA) following the manufacturer’s protocol. Briefly, cells were cultured to the logarithmic growth phase and then cross-linked with 1% formaldehyde for 10 minutes at room temperature. A glycine stop solution was added to terminate the fixation reaction. The cells were then washed with PBS and homogenized in lysis buffer with protease inhibitors. Chromatin was digested with micrococcal nuclease to yield DNA fragments ranging from 200 to 1000 base pairs. After centrifugation, the supernatant was used for chromatin immunoprecipitation with control IgG or a specific antibody to β-catenin (Cell Signaling Technology, Cat. No. D10A8). Input samples consisted of 10 µL of sheared chromatin. For each ChIP reaction, 10 µg of sheared chromatin was combined with 3 µg of specific antibody. The chromatin-antibody mixture was incubated overnight at 4 °C on a rotator. Protein G magnetic beads and protease inhibitors were added during incubation to facilitate immunoprecipitation. ChIP and input DNA samples were de-crosslinked, treated with proteinase K, and purified. β-catenin occupancy of the α-promoter was analyzed by qPCR using a specific primer [31].

### In vivo xenograft model

All animals were purchased from Charles River Laboratories (Beijing, China) and maintained under specific pathogen-free conditions. For tumor xenografts, 5 × 10^6 cells were subcutaneously injected into the backs of 5-week-old NOD/Scid male nude mice. After 60 days, the NOD/Scid mice were sacrificed, and tumor tissues were harvested, cut into approximately 2 mm³ pieces, and subcutaneously implanted into the right abdomens of 5-week-old BALB/C-Nude male mice. After 14 days, BALB/C-Nude mice with comparable tumor sizes were randomly divided into two groups (DMSO vs. DCLK1-IN1). Eight mice per group received DMSO or DCLK1-IN-1 by intragastric administration every other day. Tumor volume (Volume = (Length × Width²) / 2) was measured every 4 days. Sixteen days after intragastric administration, the mice were sacrificed, photographed, and the xenografted tumors were collected for RT-qPCR and immunohistochemistry (IHC) assays. All animal experiments were approved by the Institutional Animal Care and Use Committee of Capital Medical University (Beijing, China).

### Immunohistochemistry

Tumor tissues were fixed in 4% paraformaldehyde (Solarbio, Beijing, China), embedded in paraffin, and sliced into 4 µm-thick sections for IHC analysis. Briefly, the tissue sections were deparaffinized in xylene for 30 minutes and hydrated in a graded alcohol series (100%, 95%, and 80% ethanol) for 5 minutes each. After antigen retrieval with EDTA (ZSGB-BIO, China) and blocking with goat serum for 1 hour at room temperature, the sections were incubated with the appropriate antibody at 4 °C overnight. Following incubation with enzyme-labeled goat anti-mouse/rabbit IgG polymer (ZSGB-BIO, China), the IHC signal was visualized using 3,3’-diaminobenzidine (DAB, ZSGB-BIO, China) and captured under a light microscope (Olympus Corporation, Tokyo, Japan) at 200x magnification.

### Bioinformatic analysis

High-throughput sequencing data (HTSeq) and clinical information from TCGA were downloaded from UCSC Xena (https://xenabrowser.net/datapages/). Fragments Per Kilobase Million (FPKM) values were log-transformed to achieve a normal distribution (log2 (FPKM + 1)) for subsequent statistical analyses. The Wilcoxon rank-sum test was applied to compare DCLK1 expression levels between cancer and adjacent normal tissues. To compare relative isoform percentages between PLOD2-low and PLOD2-high ccRCCs, TCGA ccRCC samples were split into high and low groups based on the optimal cut-off for PLOD2 FPKM data, determined using the “surv_cutpoint” function in the “survminer” R package. The correlation between PLOD2 and DCLK1 isoform 1/2 expression was assessed using the Pearson correlation test. A heatmap was generated using the “ComplexHeatmap” R package to illustrate the relationship between the PLOD2-DCLK1-L axis and the EMT gene signature. For survival analysis, ccRCC patients were divided into high and low groups based on optimal cut-off values for DCLK1-L and PLOD2, determined using the “survminer” R package. The 12-year disease-free intervals (DFI) of these groups were then statistically compared using Kaplan-Meier curves and log-rank tests with the “survival” and “survminer” R packages. PSI values for splice events in tumor samples were analyzed using TCGA SpliceSeq (http://projects.insilico.us.com/TCGASpliceSeq/about.jsp) [39].

### Statistical analysis

Data analysis was conducted using GraphPad Prism 7.0 (GraphPad Software, RRID: SCR_002798) unless otherwise specified. For parametric analyses, Student’s t-test was used to determine statistical significance. For non-parametric analyses, the Mann-Whitney test was applied to assess statistical significance. Fisher’s exact test was used for categorical data analysis. Kaplan-Meier curves and log-rank tests were used to evaluate differences in survival times between groups. Quantitative data are presented as mean ± standard deviation. Statistical significance was defined as *p* < 0.05. Levels of significance were indicated as follows: *p < 0.05; **p < 0.01; ***p < 0.001; ****p < 0.0001.

## Results

### Hyperactivation and alternative-promoter switching of DCLK1 towards the long- isoforms in ccRCC

**Fig. 1:**
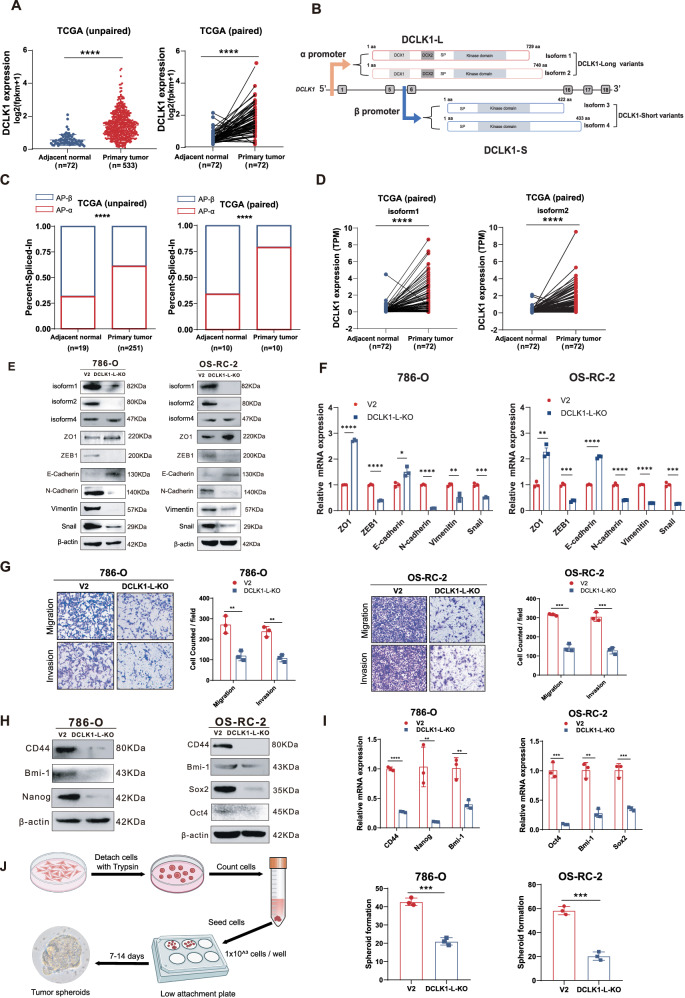
Hyperactivation and alternative-promoter switch of DCLK1 towards the long isoform in ccRCC tumorigenesis. **A** Comparisons of DCLK1 mRNA expression between adjacent normal and primary tumor in the TCGA ccRCC dataset. Left, unpaired analysis; right, paired analysis. **B** A diagram illustrating the usage of alternative promoters of the DCLK1 to generate long and short isoforms. **C** Comparisons of the PSI values of AP-α and AP-β between adjacent normal and primary tumor in the TCGA ccRCC dataset. Left, unpaired analysis; right, paired analysis. **D** Comparisons of the expression of DCLK1-Long isoform1 and isoform 2 between primary tumor and paired normal tissues in the TCGA ccRCC dataset. **E** Western blotting assays to confirm the specific deletion of DCLK1-L and the effect of DCLK1-L silence on EMT markers in 786-O (left) and OS-RC-2 cells (right) at the protein level. **F** RT-qPCR assays to examine the effect of DCLK1-L deletion on EMT markers at the mRNA level. **G** Transwell assays to examine the effect of DCLK1-L deletion on cell migration and invasion properties in 786-O and OS-RC-2 cells. **H** Western blotting examination of the effect of DCLK1-L deletion on the protein level of cancer stem cell markers in 786-O and OS-RC-2 cells. **I** RT-qPCR determination of the mRNA level of the cancer stem cell markers in 786-O and OS-RC-2 cells. (**J**) Representative images and statistical bar charts showing the effect of DCLK1-L deletion on cancer stem cell spheroid-forming abilities of 786-O and OS-RC-2 cells. The data are presented as the mean ± SD from three independent experiments performed in triplicate. *p < 0.05, **p < 0.01, ***p < 0.001, ****p < 0.0001. DCLK1-L, DCLK1 long isoforms; V2, LentiCRISPRV2 control vector.

To assess DCLK1 expression in ccRCC, we analyzed TCGA data. Both unpaired and paired analyses showed a consistent and significant upregulation of DCLK1 in ccRCC compared to adjacent noncancerous tissue (Fig. 1A). Given the reported variability in expression and function of DCLK1 long and short isoforms across different cancer types, we further explored the dominant oncogenic isoform in ccRCC. Since DCLK1-L and DCLK1-S isoforms are generated respectively from the alternative usage of α and β promoters (Fig. 1B), we evaluated changes in DCLK1 alternative-promoter choice during ccRCC tumorigenesis. We performed SpliceSeq analysis [39] to compare changes in the two AP events between ccRCC and adjacent noncancerous samples in TCGA. For clarity, AP events on the α-promoter are designated as AP-α and those on the β-promoter as AP-β. Based on Percent Spliced In (PSI) values of the two mutually exclusive AP events in the TCGA KIRC database, the α- and β-promoters are initiated with probabilities of 31.6% and 68.4%, respectively, in adjacent noncancerous kidney tissue. In ccRCC, the α-promoter is initiated with an elevated probability of 61.1%, while that of the β-promoter declines to 38.9% (Fig. 1C, left). A similar trend was also observed in paired sample analysis (α-promoter: 34.15% vs. 65.85%; β-promoter: 79.03% vs. 20.97%) (Fig. 1C, right). These data suggest a shift in alternative-promoter choice from β to α during ccRCC tumorigenesis. Given that AP regulation is often tissue-specific [40], we compared this alteration in ccRCC with other cancer types for which PSI values are available for both tumor and normal samples in the TCGA SpliceSeq database. The results showed that the β-promoter is more frequently activated in normal kidney compared to other normal tissues, and that the β-to-α promoter shift is more pronounced in ccRCC (Fig. S1). These data suggest potential oncogenic activity for the α-promoter in ccRCC. In accordance, TCGA analysis showed that both α-promoter-initiated DCLK1-L variants (isoform 1 and 2) are significantly increased in ccRCC compared to the adjacent normal tissue (Fig. 1D).

### DCLK1- L confers cancer stemness and aggressiveness in ccRCC

To assess the oncogenic role of DCLK1-L in ccRCC, we performed loss-of-function studies by CRISPR/Cas9-mediated specific depletion of DCLK1-L variants in 786-O and OS-RC-2 ccRCC cell lines using DCLK1-L specific small guide RNAs. Efficient deletion of DCLK1-L isoforms was confirmed at the protein level using Western blotting (Fig. 1E), while DCLK1-S expression remained unaffected, as indicated by isoform 4 levels (Fig. 1E). DCLK1-S isoform 3 has been reported to be absent or expressed at extremely low levels in ccRCC [23] and was barely detectable in our experiments.

Given the well-known function of DCLK1 in provoking EMT in cancer [25, 41, 42], we examined the role of DCLK1-L in EMT in ccRCC. We found that silencing DCLK1-L led to significant EMT inactivation, as evidenced by marked decreases in mesenchymal markers, including N-cadherin, Vimentin, ZEB-1, Snail and Slug, along with increased expression of epithelial markers E-cadherin and ZO-1, in 786-O and OS-RC-2 cells (Fig. 1E). EMT inactivation was further confirmed by RT-qPCR in both 786-O and OS-RC-2 cells (Fig. 1F). In line with EMT inactivation, transwell assays demonstrated significantly impaired migration and invasion in DCLK1-L knockout ccRCC cell lines compared to control cells (Fig. 1G). These data identify a critical role for DCLK1-L in activating EMT during ccRCC metastasis.

Additionally, as a putative CSC marker, DCLK1 is known to maintain stem cell-like properties in cancers [23, 29, 43]. We therefore further examined the effect of DCLK1-L deletion on ccRCC stemness. Western blotting (Fig. 1H) and RT-qPCR (Fig. 1I) results showed that DCLK1-L knockout decreased the expression of known CSC markers and pluripotency factors, including CD44, Bmi-1, Nanog, Sox2, and Oct4, in 786-O and OS-RC-2 cells. Furthermore, spheroid formation assays confirmed that depletion of DCLK1-L reduced functional CSC properties in ccRCC (Fig. 1J). Collectively, the above findings highlight the functional importance of DCLK1-L in maintaining EMT activation and stemness in ccRCC.

### Hypoxia signaling governs DCLK1 activation and α-promoter preference in a HIF2α-dependent manner

**Fig. 2:**
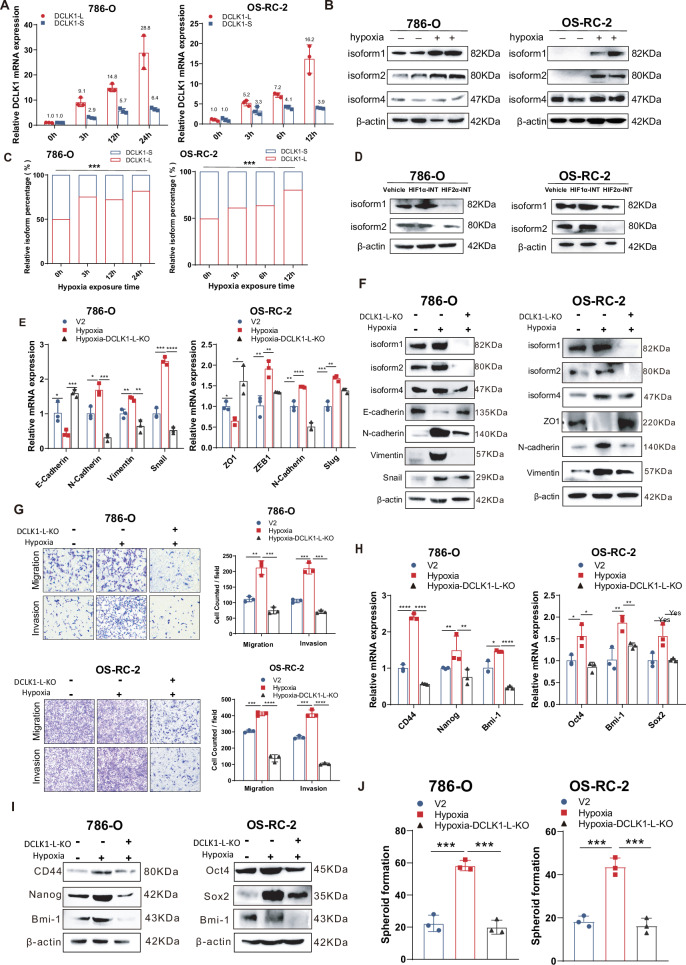
Hypoxia signaling governs DCLK1 activation and α-promoter preference in a HIF2α dependent manner, and DCLK1-L mediates hypoxia-activated cancer stemness and aggressiveness in ccRCC. **A** RT-qPCR examination of the effect of hypoxia exposure (0, 3, 6, 12, and 24 h) on the mRNA expression of DCLK1-L and DCLK1-S in 786-O and OS-RC-2 cells. **B** Western blotting assays to detect the effect of hypoxia on the protein levels of DCLK1-L (isoform1 and isoform2) and DCLK1-S (isoform4) in 786-O and OS-RC-2 cells. **C** RT-qPCR determination of the relative isoform percentages of DCLK1-L and DCLK1-S at different time points under hypoxia. **D** Western blotting assays to examine the effect of HIF1α-INT and HIF2α-INT treatment on the expression of DCLK1-L at the protein level. **E** RT-qPCR measurement of the changes in the relative mRNA level of EMT markers in the control and DCLK1-L deleted ccRCC cells when exposed to hypoxia for 48 h. **F** Western blotting assays to determine the changes in the relative protein level of EMT markers in the control and DCLK1-L deleted ccRCC cells upon hypoxia exposure. **G** Transwell assays to examine the effect of DCLK1-L deletion on hypoxia-driven migration and invasion in two cell lines. **H** RT-qPCR assays to assess the effect of DCLK1-L silencing on hypoxia-activated CSC markers at the mRNA level in two cancer cell lines. **I** Western blotting assays to examine the effect of DCLK1-L silencing on hypoxia-activated CSC markers at the protein level. **J** Representative images and statistical bar charts showing the effect of DCLK1-L deletion on hypoxia-driven tumor spheroids-formation ability. The data are presented as the mean ± SD from three independent experiments performed in triplicate. *p < 0.05, **p < 0.01, ***p < 0.001, ****p < 0.0001. HIF1α-INT: the HIF-1α-Inhibitor BAY87-2243; HIF2α-INT: the HIF-2α-Inhibitor PT2385.

Having identified the oncogenic contribution of DCLK1-L in ccRCC, we sought to further investigate the mechanism underlying DCLK1 activation and alternative promoter (AP) model switching in ccRCC. Specifically, we aimed to determine if the β-to-α AP model switch was regulated by hypoxia signaling, a characteristic feature of ccRCC. To test this hypothesis, we treated ccRCC cell lines with hypoxia and observed an additive activation of HIF2α even in the pVHL-inactivated 786-O and OS-RC-2 cell lines, both of which have with abundant constitutive HIF2α (Fig. S2), consistent with previous reports [32, 33]. Subsequently, changes in DCLK1-L and DCLK1-S levels were examined by RT-qPCR using isoform-specific primers. Results showed that hypoxia exposure caused a robust upregulation of DCLK1-L (9.1, 14.8, and 28.8 fold at 3, 12, and 24 h post-treatment respectively) but much less elevation of DCLK1-S (2.9, 5.7, and 6.4 fold) in 786-O cells (Fig. 2A, left), which was also verified in OS-RC-2 cells (Fig. 2A, right). Preferential activation of DCLK1-L was further confirmed at the protein level in both cell lines, while DCLK1 isoform 4 showed no significant change (Fig. 2B). The stepwise increase in DCLK1-L percentage with longer hypoxia exposure demonstrated progressive α-promoter usage switching during hypoxia treatment (Fig. 2C). These findings indicate that hypoxia drives α-promoter-preferred DCLK1 activation.

The alpha subunits of the hypoxia-inducible transcription factors (HIF1α and HIF2α) play pivotal roles in mediating the hypoxia response [44, 45]. In contrast to most tumor types, HIF1α in ccRCC is often diminished by chromosomal deletion and acts as a tumor suppressor, while HIF2α serves as the primary mediator of hypoxia signaling and the main driver of ccRCC biology [46]. Consistent with this notion, RT-qPCR assays showed that targeting HIF2α in pVHL-null 786-O and OS-RC-2 cells by the specific inhibitor PT2385 resulted in a significant reduction in DCLK1-L expression, whereas inhibiting HIF1α did not result in a notable decrease (Fig. S3). This finding was further validated at the protein level by examination of DCLK1 isoform 1 and isoform 2 (Fig. 2D), suggesting that HIF2α is the key regulator of DCLK1-L activation in ccRCC. This conclusion is further supported by hypoxia-activation of DCLK1-L expression in 786-O cells, where functional HIF1α is innately lost due to gene truncation.

### DCLK1-L mediates hypoxia-activated cancer stemness and aggressiveness in ccRCC

Hypoxia signaling enhances EMT and stem cell-like properties in cancer cells [47]. Given that DCLK1-L is a downstream target of hypoxia and promotes cancer stemness and aggressiveness in ccRCC, we investigated whether DCLK1-L is essential for hypoxia-induced malignancy in this context.

To test this, control and DCLK1-L-depleted 786-O and OS-RC-2 cells were treated with hypoxia, and the effect of DCLK1-L deletion on hypoxia response was examined. As expected, measurement of EMT markers by RT-qPCR (Fig. 2E) and Western blotting (Fig. 2F) in control cells showed clear downregulation of epithelial markers and upregulation of mesenchymal markers, indicative of EMT activation under hypoxia. However, DCLK1-L ablation substantially diminished hypoxia-induced EMT-related expression changes in both 786-O and OS-RC-2 cell lines (Fig. 2E, F). Consistently, transwell assays demonstrated that DCLK1-L knockout significantly reduced hypoxia-induced cell migration and invasion in both ccRCC cell lines (Fig. 2G). Furthermore, DCLK1-L depletion inhibited hypoxia-induced CSC marker expression, as determined by RT-qPCR (Fig. 2H) and Western blotting (Fig. 2I). Functionally, this was accompanied by a significant reduction in tumor spheroid formation (Fig. 2J). These findings indicate that hypoxia-induced DCLK1-L expression is essential for maintaining hypoxia-induced stemness and aggressiveness in ccRCC.

### PLOD2 activates DCLK1 in an α-promoter-preferred manner

**Fig. 3:**
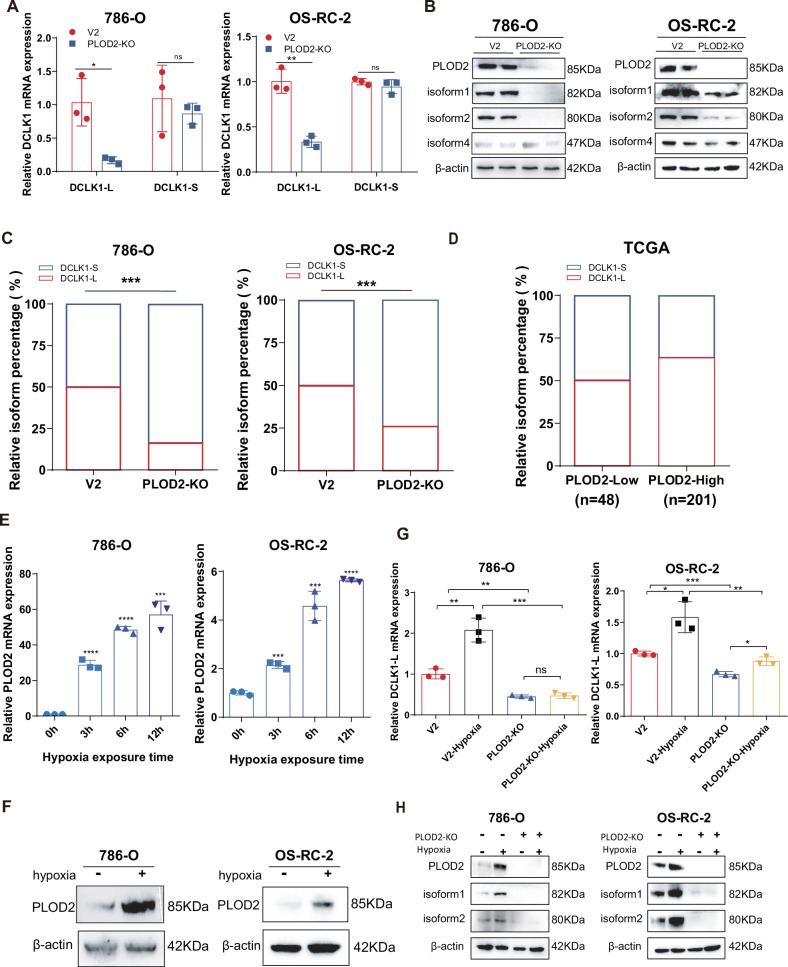
HIF2α-downstream PLOD2 mediates hypoxia preferential activation of DCLK1-L. **A** RT-qPCR assays to assess the effect of PLOD2 ablation on the mRNA level of DCLK1-L and DCLK1-S in 786-O and OS-RC-2 cells. **B** Western blotting assays to examine the effect of PLOD2 deletion on the protein level of DCLK1 long and short variants in 786-O and OS-RC-2 cells. **C** RT-qPCR comparison of the relative percentage of DCLK1-L and DCLK1-S in the control and PLOD2-deficient ccRCC cells. **D** TCGA analysis showing the relative percentage change of DCLK1-L and DCLK1-S isoforms in PLOD2-low and PLOD2-high ccRCCs. **E** RT-qPCR assays to assess the influence of hypoxia on the mRNA expression of PLOD2 in 786-O and OS-RC-2 cells. **F** Western blotting assays to examine the effect of hypoxia treatment (48 h) on PLOD2 expression at the protein level in 786-O and OS-RC-2 cells. **G** RT-qPCR assays to determine the effect of PLOD2 deletion on hypoxia-activated DCLK1-L expression at the mRNA level. **H** Western blotting assays to assess the effect of PLOD2 deletion on hypoxia-activated DCLK1-L (isoform 1 and isoform 2) expression at the protein level. The data are presented as the mean ± SD from three independent experiments performed in triplicate. *p < 0.05, **p < 0.01, ***p < 0.001; ****p < 0.0001; ns non-significant.

Recent advances have highlighted the pivotal role of PLOD2 in mediating hypoxia-driven tumor metastasis [48–50]. In an effort to decipher the mechanism underlying PLOD2-driven cancer metastasis in ccRCC, we found that PLOD2 was also essential for selective activation of DCLK1-L. CRISPR/Cas9-mediated ablation of PLOD2 substantially decreased DCLK1-L expression but did not cause an obvious change in DCLK1-S expression (isoform 4), in both 786-O and OS-RC-2 cell lines, at both the mRNA (Fig. 3A) and protein levels (Fig. 3B). Comparison of isoform relative percentages via RT-qPCR demonstrated a significant α-to-β AP usage switch following PLOD2 knockout (Fig. 3C). The role of PLOD2 in preferentially facilitating α-promoter initiation was further supported by TCGA analysis, which showed that PLOD2-high ccRCCs harbor a higher AP-α occurrence probability than PLOD2-low ccRCCs (Fig. 3D). Overall, these findings indicate a role for PLOD2 in regulating α-promoter-preferred DCLK1 activation.

### HIF2α-downstream PLOD2 mediates hypoxia activation of DCLK1-L

Given that both hypoxia and PLOD2 play critical roles in α-promoter-preferred DCLK1 activation, and that PLOD2 is a known a hypoxia-responsive gene in multiple cancers [38, 48, 51], we hypothesized that PLOD2 mediates hypoxia-induced activation of DCLK1-L. To test this, we first examined whether PLOD2 is a downstream target of hypoxia in ccRCC. Strikingly, both RT-qPCR (Fig. 3E) and Western blotting (Fig. 3F) analyses confirmed hypoxia-induced activation of PLOD2 in 786-O and OS-RC-2 cell lines. To assess the role of PLOD2 in mediating hypoxia-induced DCLK1-L activation, control and PLOD2-depleted ccRCC cells were exposed to hypoxia, and DCLK1-L expression was examined by RT-qPCR. Results showed that PLOD2 deficiency substantially abrogated hypoxia-activated DCLK1-L expression at the mRNA level (Fig. 3G), and this was further validated at the protein level by Western blotting in PLOD2-depleted 786-O and OS-RC-2 cells (Fig. 3H). These findings establish an essential role for PLOD2 in mediating hypoxia-induced DCLK1-L activation.

Since both PLOD2 and HIF2α are crucial for hypoxia-activated DCLK1-L, we further investigated the relationship between HIF2α and PLOD2 in mediating the hypoxia-DCLK1 signaling. We found that, in parallel to the aforementioned effect of HIF inhibition on DCLK1-L, HIF2α inhibition led to greater suppression of PLOD2 than HIF1α inhibition in both cell lines, validated at both the mRNA and protein levels (Fig. S4A). PLOD2 knockout had no significant effect on HIF1α or HIF2α (Fig. S4B), suggesting that HIF2α functions upstream of PLOD2 in hypoxia signaling. Collectively, these findings reveal a hypoxia-HIF2α-PLOD2 signaling axis that preferentially activates DCLK1-L in ccRCC.

### The hypoxia-PLOD2 axis preferentially drives DCLK1-L activation via β-catenin

**Fig. 4:**
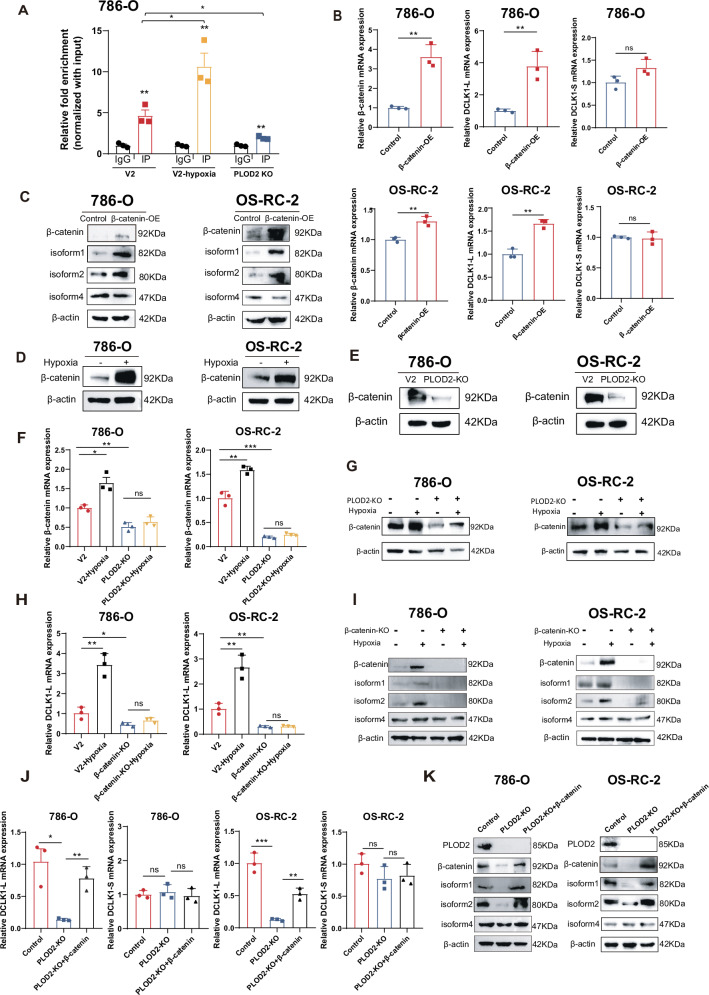
The hypoxia-HIF2α-PLOD2 axis preferentially drives DCLK1-L activation via β-catenin. **A** ChIP-qPCR assay to determine the binding of β-catenin to the DCLK1 α-promoter. **B**, **C** RT-qPCR (**B**) and Western blotting (**C**) assays to examine the effect of β-catenin overexpression on the expression of DCLK1-L and DCLK1-S in 786-O and OS-RC-2 cells. **D** Western blotting assays to evaluate the effects of hypoxia on the expression of β-catenin in 786-O and OS-RC-2 cells. **E** Western blotting assays to evaluate the effects of PLOD2 deletion on the expression of β-catenin in 786-O and OS-RC-2 cells. **F**, **G** RT-qPCR (**F**) and Western blotting (**G**) assays to determine the effect of PLOD2 silencing on hypoxia-activated β-catenin in 786-O and OS-RC-2 cells. **H** RT-qPCR assays to assess the effect of β-catenin deletion on hypoxia-activated DCLK1-L in 786-O and OS-RC-2 cells. **I** Western blotting assays to examine the effect of β-catenin silencing on hypoxia-induced expression of DCLK1-L (isoform1, isoform2) and DCLK1-S (isoform4) in 786-O and OS-RC-2 cells. **J** RT-qPCR assays to assess the effect of β-catenin restoration on PLOD2 deletion-impaired DCLK1-L expression in 786-O and OS-RC-2 cells. **K** Western blotting assays to examine the effect of β-catenin restoration on the expression of DCLK1-L (isoform1, isoform2) and DCLK1-S (isoform4) in PLOD2-depleted ccRCC cells. The data are presented as the mean ± SD from three independent experiments performed in triplicate. *p < 0.05, **p < 0.01, ***p < 0.001; ns non-significant.

β-catenin is well-known in promoting cancer stemness and EMT, and has been reported to activate DCLK1-L transcription by binding to TCF4/LEF elements within the α-promoter [31]. Therefore, we tested whether the hypoxia-PLOD2 axis preferentially activates DCLK1-L expression via β-catenin. We validated β-catenin’s α-promoter binding activity by ChIP-qPCR in ccRCC (Fig. 4A) and further confirmed its capacity to selectively activate the α-promoter of DCLK1, as evidenced by the preferential upregulation of DCLK1-L over DCLK1-S upon β-catenin overexpression at the mRNA (Fig. 4B) and protein (Fig. 4C) levels in both 786-O and OS-RC-2 cell lines. These data in ccRCC cells further verified the previously reported function of β-catenin in selective activation of DCLK1-L [31].

To evaluate whether β-catenin is downstream of the hypoxia-HIF2α-PLOD2 axis, we examined the effects of hypoxia exposure and PLOD2 inhibition on β-catenin expression. As expected, hypoxia robustly induced β-catenin expression (Fig. 4D), while PLOD2 depletion substantially reduced it (Fig. 4E) in both 786-O and OS-RC-2 cell lines. Furthermore, PLOD2 knockout significantly abrogated hypoxia-induced β-catenin expression at both the mRNA (Fig. 4F) and protein levels (Fig. 4G) in both ccRCC cell lines. These findings identify β-catenin as a downstream target of the hypoxia-HIF2α-PLOD2 axis.

To assess the role of β-catenin in mediating DCLK1-L activation by the hypoxia-HIF2α-PLOD2 axis, we first performed ChIP-qPCR analysis and found that hypoxia exposure significantly increased β-catenin’s occupancy of the α-promoter, while PLOD2 depletion significantly decreased it (Fig. 4A), indicating that the hypoxia-HIF2α-PLOD2 axis promotes β-catenin binding to the α-promoter. To further confirm that the hypoxia-HIF2α-PLOD2 axis selectively activates DCLK1-L via β-catenin, we next evaluated β-catenin’s role in hypoxia- and PLOD2-regulated DCLK1-L expression. Strikingly, RT-qPCR and Western blotting in both ccRCC cell lines revealed that β-catenin deficiency diminished hypoxia-induced DCLK1-L activation (Fig. 4H, I), while β-catenin restoration rescued PLOD2 depletion-suppressed DCLK1-L expression (Fig. 4J, K). As expected, no apparent alterations in DCLK1-S expression (isoform 4) were detected (Fig. 4I, K), further highlighting that the hypoxia-HIF2α-PLOD2 axis preferentially regulates DCLK1-L over DCLK1-S via β-catenin.

### PLOD2 mediates hypoxia-activated cancer stemness and invasiveness

**Fig. 5:**
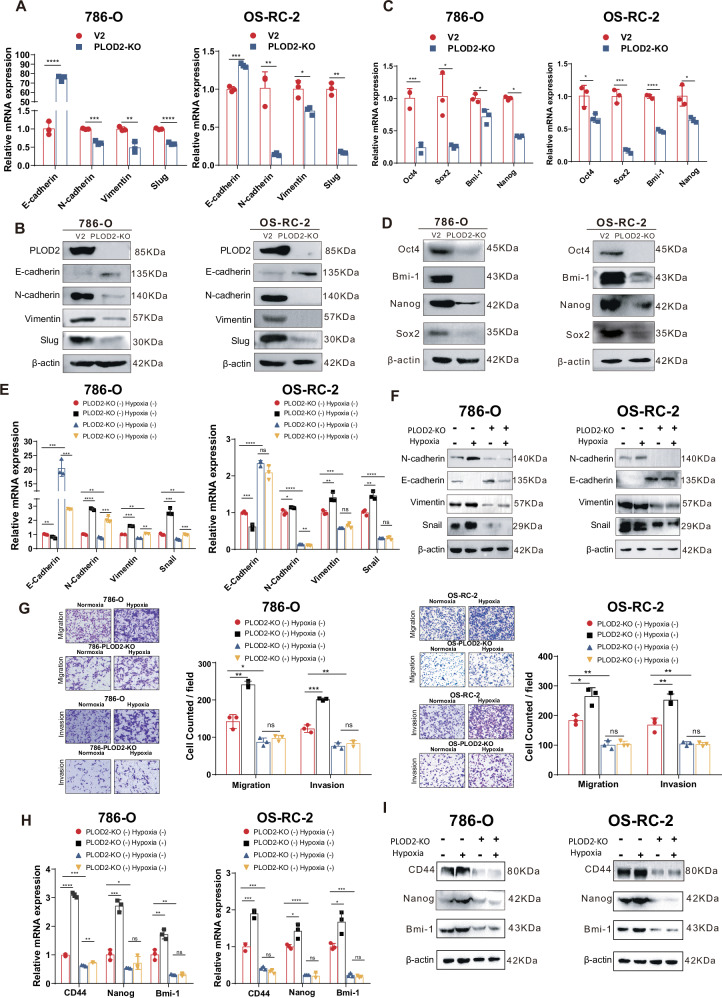
PLOD2 mediates hypoxia-activated cancer stemness and invasiveness. **A** RT-qPCR assays to determine the effect of PLOD2 deletion on the mRNA level of EMT markers in 786-O and OS-RC-2 cells. **B** Western blotting assays to assess the effect of PLOD2-deletion on the protein level of EMT markers in 786-O and OS-RC-2 cells. **C** RT-qPCR assays to examine the effect of PLOD2 ablation on the mRNA level of CSC markers in 786-O and OS-RC-2 cells. **D** Western blotting assays to show the effect of PLOD2 ablation on the protein level of CSC markers in 786-O and OS-RC-2 cells. **E** RT-qPCR assays to examine the effect of PLOD2 deletion on the mRNA level of hypoxia-activated EMT markers in 786-O and OS-RC-2 cells. **F** Western blotting assays to assess the effect of PLOD2 silencing on the protein level of hypoxia-activated EMT markers in 786-O and OS-RC-2 cells. **G** Transwell assays to examine the effect of PLOD2 deletion on hypoxia-driven migration and invasion in 786-O and OS-RC-2 cells. **H** RT-qPCR assays to examine the effect of PLOD2 deficiency on the mRNA level of CSC markers in 786-O and OS-RC-2 cells. **I** Western blotting assays to assess the effect of PLOD2 deficiency on the protein level of CSC markers in 786-O and OS-RC-2 cells. The data are presented as the mean ± SD from three independent experiments performed in triplicate. *p < 0.05, **p < 0.01, ***p < 0.001, ****p < 0.0001; ns non-significant.

Having identified PLOD2’s pivotal role in transducing the hypoxia-DCLK1-L cascade, we hypothesized that it has a critical functional contribution to this signaling. To test this hypothesis, we first examined the role of PLOD2 alone in ccRCC and found that PLOD2 deletion led to a notable loss of EMT molecular characteristics (Fig. 5A, B) and a reduction in CSC markers (Fig. 5C, D) at both the mRNA and protein levels in 786-O and OS-RC-2 ccRCC cell lines. Moreover, PLOD2 knockout effectively blocked hypoxia-induced EMT activation (Fig. 5E, F), and prevented hypoxia from triggering cancer cell migration and invasion in transwell assays in both cell lines (Fig. 5G). Similarly, PLOD2 depletion abrogated hypoxia-induced acquisition of stem-like molecular properties in 786-O and OS-RC-2 cells as determined by RT-qPCR (Fig. 5H) and Western blotting (Fig. 5I).

### Both DCLK1-L variants are essential for hypoxia-PLOD2-driven invasiveness and stemness

**Fig. 6:**
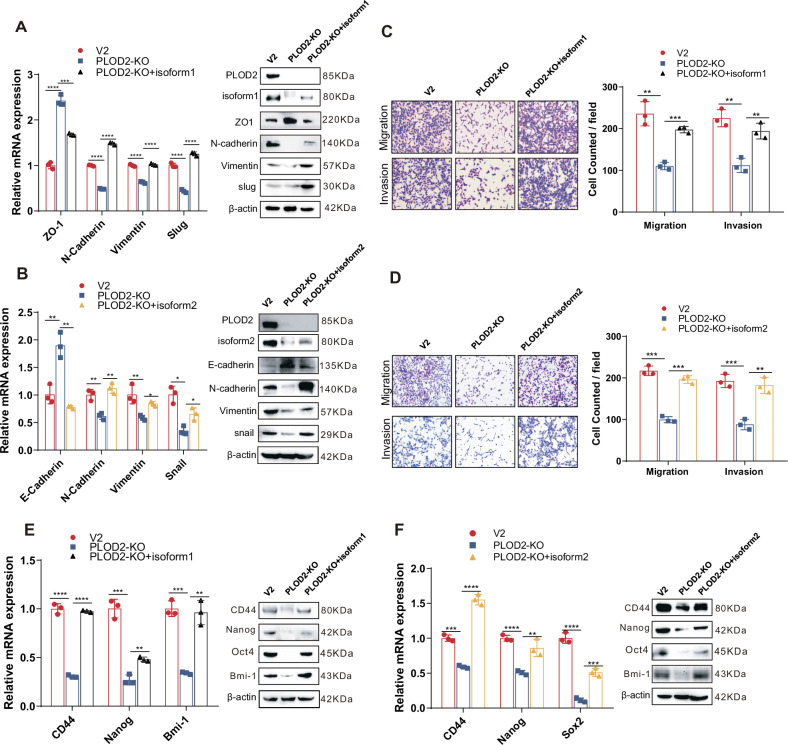
Both DCLK1-L variants are essential for hypoxia-PLOD2-driven cancer invasiveness and stemness. **A** RT-qPCR and Western blotting assays to evaluate the effect of DCLK1-isoform1 restoration on PLOD2 deficiency-suppressed EMT markers expression in 786-O cells. **B** RT-qPCR and Western blotting assays to evaluate the effect of DCLK1-isoform2 restoration on PLOD2 deficiency-suppressed EMT markers expression in 786-O cells. **C**, **D** Transwell analyses to examine the effect of DCLK1-isoform1 (**C**) and DCLK1-isoform 2 (**D**) restoration on PLOD2-deletion-impaired migration and invasion in 786-O cells. **E**, **F** RT-qPCR and Western blotting assays to assess the effect of DCLK1-isoform1 (**E**) and DCLK1-isoform 2 (**F**) restoration on PLOD2-deletion-suppressed CSC markers expression. The data are presented as the mean ± SD from three independent experiments performed in triplicate. *p < 0.05, **p < 0.01, ***p < 0.001, ****p < 0.0001.

The functional importance of both DCLK1-L and PLOD2 in hypoxia-driven ccRCC malignance, along with the validated PLOD2-DCLK1-L cascade in hypoxia signaling, prompted us to further examine the contribution of DCLK1-L to PLOD2-driven cancer invasiveness and stemness. To test this, we rescued DCLK1-L expression in PLOD2-KO ccRCC cells and assessed its effects on malignant properties suppressed by PLOD2 deficiency. To further distinguish the functional differences between the two DCLK1-L variants, DCLK1 isoform 1 and isoform 2 vectors were individually transfected into PLOD2-deleted ccRCC cells. Restoration of either DCLK1 isoform 1 (Fig. 6A) or isoform 2 (Fig. 6B) rescued impaired EMT properties in PLOD2-KO ccRCC cells and restored their migratory and invasive abilities (Fig. 6C, D). Both DCLK1 isoform 1 (Fig. 6E) and isoform 2 (Fig. 6F) also CSC marker expression suppressed by PLOD2 deficiency. Together, these results identify a HIF2α-PLOD2-β-catenin-DCLK1-L signaling axis that governs the induction of metastatic and stemness properties in ccRCC.

### Hyperactivation of the PLOD2-DCLK1-L axis is associated with an unfavorable prognosis in ccRCC

**Fig. 7:**
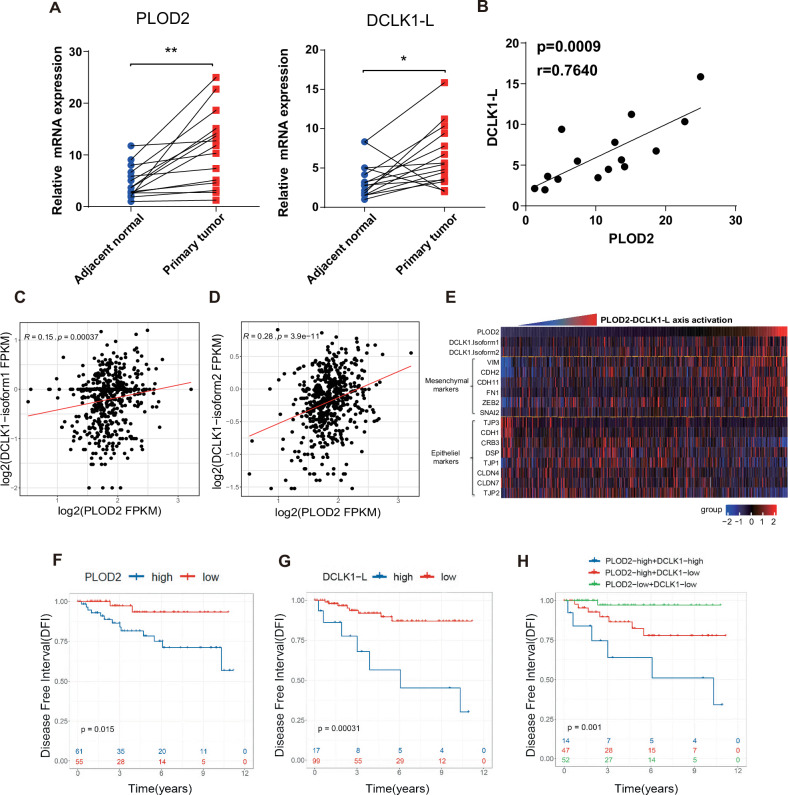
Hyperactivation of the PLOD2-DCLK1-L axis is associated with an unfavorable prognosis in ccRCC. **A** RT-qPCR assays to examine the levels of PLOD2 and DCLK1-L in ccRCC and matched adjacent normal tissues in a ccRCC cDNA microarray (*n* = 15 for each group). **B** Pearson’s correlation analysis to determine the correlation of PLOD2 and DCLK1-L in the tumor samples of the cDNA microarray. **C**, **D** Pearson’s correlation analysis showing the positive correlation of PLOD2 and DCLK1-isofrom1 (**C**) or DCLK1-isoform2 (**D**) in the TCGA ccRCC dataset. **E** Heatmap clustering analysis showing the correlation of the PLOD2-DCLK1-L axis and EMT gene signature in the ccRCC patients in the TCGA dataset. **F** Kaplan–Meier analysis showing the association between PLOD2 level and disease-free interval (DFI) of ccRCC patients in the TCGA database. **G** Kaplan–Meier analysis showing the association between DCLK1-L level and DFI of ccRCC patients in the TCGA database. **H** Kaplan–Meier analysis showing the relationship between the level of PLOD2-DCLK1-L axis and DFI of ccRCC patients in the TCGA database.*p < 0.05, **p < 0.01.

The overwhelming prevalence of hypoxia signaling activation in ccRCC led us to hypothesize a hyperactivated status of the PLOD2-DCLK1-L axis in this cancer type. To test this hypothesis, we measured the mRNA levels of PLOD2 and DCLK1-L using RT-qPCR in a cDNA microarray containing paired samples from 15 ccRCC patients. Results showed that PLOD2 and DCLK1-L were significantly upregulated in ccRCC samples compared to their paired normal samples (Fig. 7A). Additionally, a strong correlation was detected between PLOD2 and DCLK1-L in the tumor samples of this ccRCC cohort (r = 0.7640, *p* = 0.0009, Fig. 7B), which was further supported by significant coexpression of PLOD2 and DCLK1 isoform 1 (Fig. 7C) and isoform 2 (Fig. 7D) in the TCGA ccRCC cohort. Furthermore, activation of this axis correlated closely with an enhanced EMT signature, as shown by heatmap clustering analysis in the TCGA ccRCC dataset (Fig. 7E). Prognostically, high levels of both PLOD2 (*p* = 0.015, Fig. 7F) and DCLK1-L (*p* = 0.00031, Fig. 7G) predicted poorer disease-free survival (DFS) in the TCGA ccRCC cohort. Furthermore, the ccRCC subpopulation with high levels of both PLOD2 and DCLK1-L (PLOD2 ^high^ DCLK1-L^high^ group) showed the worst DFS, compared to improved DFS in the PLOD2 ^high^ DCLK1-L^low^ group and the best DFS in the PLOD2 ^low^ DCLK1-L ^low^ group (Fig. 7H). These results indicate that activation of the PLOD2-DCLK1-L axis is associated with poor prognosis and that targeting this axis through DCLK1-L inhibition may improve patient outcomes.

### Pharmacological targeting of DCLK1 attenuates the malignancy of hypoxic PLOD2-rich ccRCCs in vitro and in vivo

**Fig. 8:**
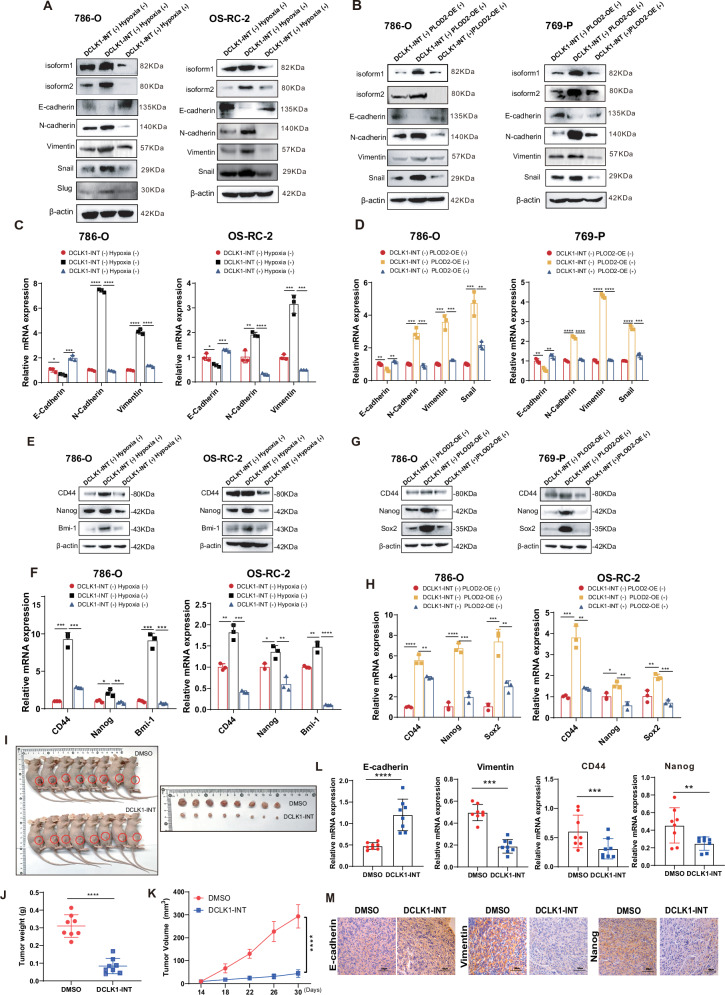
Pharmacologically targeting DCLK1 attenuates the malignance of hypoxic PLOD2-rich ccRCCs in vitro and in vivo. **A** Western blotting examination of the effect of DCLK1-INT on reversing hypoxia-activated DCLK1-L expression and EMT activation at the protein level in 786-O and OS-RC-2 cells. **B** Western blotting examination of the effect of DCLK1-INT on reversing PLOD2 overexpression-activated DCLK1-L expression and EMT activation at the protein level in 786-O and OS-RC-2 cells. **C** RT-qPCR assays to examine the effect of DCLK1-INT on hypoxia-activated EMT at the mRNA level in 786-O and OS-RC-2 cells. **D** RT-qPCR assays to examine the effect of DCLK1-INT on PLOD2 overexpression-activated EMT at the mRNA level in 786-O and OS-RC-2 cells. **E**, **F** Western blotting (**E**) and RT-qPCR (**F**) assays to show the effect of DCLK1-INT on hypoxia-driven cancer stemness in 786-O and OS-RC-2 cells. **G**, **H** Western blotting (**G**) and RT-qPCR (**H**) assays to demonstrate the effect of DCLK1-INT on PLOD2 overexpression-driven cancer stemness in 786-O and OS-RC-2 cells. **I** Photographs showing the size of tumors in DMSO and DCLK1-INT groups. **J** Comparison of the tumor weights between DMSO and DCLK1-INT groups. **K** Tumor growth curves showing the in vivo growth of tumors in DMSO and DCLK1-INT groups. **L** RT-qPCR examination of the expression of EMT and CSC markers in tumors of DMSO and DCLK1-INT groups. **M** IHC examination of the expression of EMT and CSC markers in tumors of DMSO and DCLK1-INT groups. The data are presented as the mean ± SD from three independent experiments performed in triplicate. *p < 0.05, **p < 0.01, ***p < 0.001, ****p < 0.0001.

Next, we evaluated the potential clinical benefits of pharmacological inhibition of this signaling. DCLK1 is a pharmacologically actionable target in this signaling, and the inhibitor DCLK1-IN-1 was selected for its ability to efficiently suppress DCLK1 expression [43, 52, 53] and phosphorylation [54]. Notably, DCLK1-IN-1 significantly reduced DCLK1-L induction under hypoxia (Figs. S5A and 8A) as well as with PLOD2 overexpression (Figs. S5B and 8B), thereby abrogating hypoxia- and PLOD2-induced EMT (Fig. 8A–D), migration, invasion (Fig. S5C and S5D), and stem cell-like properties (Fig. 8E–H) in vitro.

We then assessed the effect of DCLK1 inhibition on PLOD2-rich ccRCC xenografts in vivo through systemic administration of DCLK1-IN-1. The 786-O cell line, selected for its abundant PLOD2 expression and tumorigenic competence (Fig. S6), was subcutaneously injected into the NOD/Scid mice to form the PLOD2-rich ccRCC xenografts. The large xenograft tumors were the dissociated and subcutaneously seeded into BALB/c-Nude mice (Fig. S7). Tumor-bearing mice were randomly assigned to either the DMSO or DCLK1-INT group (n = 8 per group). After a 16-day treatment via gavage administration (25 mg/kg, every other day), we observed a significant reduction in tumor size (Fig. 8I), tumor weight (Fig. 8J), and growth rate (Fig. 8K) in the DCLK1-IN-1 group. The inhibition rate reached 85.1% based on tumor volume calculations. Concurrently, the EMT signature and stem cell-like properties of PLOD2-rich ccRCC xenografts were significantly repressed after DCLK1-IN-1 treatment (Fig. 8L, M), as indicated by upregulation of the epithelial marker E-cadherin, downregulation of the mesenchymal marker Vimentin, and decrease in the CSC marker Nanog, determined by RT-qPCR (Fig. 8L) and IHC (Fig. 8M) analyses. These data suggest that patients with hypoxic PLOD2-rich ccRCC may derive potential clinical benefit from pharmacological inhibition of DCLK1.

## Discussion

Despite recent advances in diagnosis and treatment, advanced metastatic ccRCC, which is characteristically resistant to chemoradiotherapy, remains devastating with a 5-year overall survival of dismal 12% [1, 6]. Accumulating evidence identifies DCLK1 as a promising target for cancer therapy [10]. DCLK1 directly marks cancer stem cells in colon cancer [55], pancreatic cancer [56], and cholangiocarcinoma [57], promotes CSC-like properties and drives cancer initiation, progression, and metastatic spread [10]. In this study, we investigated DCLK1 expression in ccRCC with a focus on alternative promoter (AP) usage in the *DCLK1* gene. We identified a hypoxia- and PLOD2-linked mechanism underlying DCLK1 activation and α-promoter preference, and evaluated the therapeutic significance of targeting DCLK1-L in ccRCC. Consistent with our findings, significant activation of DCLK1 in ccRCC has also been reported in previous studies [22, 23]. Moreover, DCLK1 is overexpressed in colorectal [15, 16, 58, 59], pancreatic [20, 21, 60], hepatocellular [24], breast [18, 19], and bladder cancers [61], suggesting its widespread activation during oncogenesis.

The *DCLK1* gene transcribes long or short variants by selectively using two promoters [27, 62], with proposed differences in subcellular distribution and molecular function between DCLK1-L and DCLK1-S [10]. A comparison of promoter activation in ccRCC and adjacent normal tissue revealed that the normal kidney preferentially activates the β-promoter, whereas ccRCC preferentially activates the α-promoter, indicating an AP model switch during ccRCC tumorigenesis. This finding contrasts with previous reports in colon cancer, where most human colon cancers express DCLK1-S from the β-promoter, while normal colons primarily express DCLK1-L from the α-promoter [63–65]. Mechanistic investigations in human colon cancer revealed that the 5’ (α) promoter is hypermethylated and epigenetically silenced, resulting in preferential activation of β-promoter and predominate upregulation of DCLK1-S variants [62]. Beyond colon cancer, a hypermethylated α-promoter has also been observed in lung, pancreatic, gastric, and cholangiocarcinoma cancers [28, 29, 66, 67]. In contrast, evidence suggests that ccRCC has strong hypomethylation in the α-promoter region [23]. This unique hypomethylation in the α-promoter likely contributes to a relaxed chromatin structure that facilitates preferential α-promoter activation in ccRCC, explaining the β-to-α promoter shift in this cancer type.

The factors mediating selective use of alternative promoters in the *DCLK1* gene remain largely unknown. Le Hellard et al. reported that brain-derived neurotrophic factor (BDNF) activated the expression of DCLK1-S variants but did not affect DCLK1-L transcripts in rat hippocampus [68]. Sarkar et al. demonstrated that FOXD3 acts as a potent transcriptional repressor of the β-promoter in the human *DCLK1* gene [69]. In this study, to identify factors governing DCLK1 activation and α-promoter preference, we focused on hypoxia signaling, given the constitutive activation of hypoxia signaling in ccRCCs with VHL mutation, which accounts for over 90% of all ccRCC cases [46, 70]. We observed a significantly greater upregulation of DCLK1-L compared to DCLK1-S under hypoxia, along with a progressive increase in the DCLK1-L percentage over the course of hypoxia treatment. Few studies have focused on elucidating the interaction between DCLK1 and hypoxia regulation. To our knowledge, this is the first study to systematically examine the relationship between hypoxia signaling and DCLK1 promoter selection and activation. These findings are supported by evidence indicating that hypoxia strongly enhances DCLK1-L expression in pancreatic cancer cells [71].

PLOD2 is a pro-metastatic oncoprotein strongly implicated in mediating the hypoxia response in cancer metastasis [48, 50]. Here, we show that PLOD2 is essential for selective transcription of DCLK1-L in ccRCC, as PLOD2 silencing reversed β-to-α AP switching in both 786-O and OS-RC-2 cell lines. Furthermore, we found that PLOD2 mediates hypoxia-induced activation of DCLK1-L in ccRCC. The HIF transcription factors (HIF1α and 2α) are critical in mediating the hypoxia response [44, 45], and PLOD2 has been reported as a downstream target of HIF1α [48, 49]. However, in 786-O cells with innate HIF1α deficiency, we found that hypoxia can still efficiently activate PLOD2 and its downstream target, DCLK1-L. Further analysis using HIF1α and HIF2α inhibitors in both 786-O and OS-RC-2 cells showed that PLOD2 and DCLK1-L were preferentially suppressed by the HIF2α inhibitor. These findings suggest that HIF2α may play a more important role than HIF1α in mediating hypoxia-induced activation of PLOD2-DCLK1-L signaling in ccRCC. This may be due to the dominant expression of HIF2α in ccRCC [46, 72] or the cancer-specific functional variation between HIF1α and HIF2α in ccRCC [73]. Unlike their similar oncogenic roles in most tumors, HIF1α and HIF2α reportedly exert opposing effects on ccRCC biology, with HIF1α acting as a tumor suppressor while HIF2α acts as an oncoprotein [46, 74]. Thus, from a ccRCC-specific functional perspective, it seems rational that PLOD2 and DCLK1 function consistently downstream of HIF2α, conferring malignant properties on ccRCC. Consistent with our findings, a recent report also revealed hypoxia-induced PLOD2 expression in ccRCC cells, including 786-O [75]. However, the HIF1α-dependent mechanism proposed in this report is unconvincing for 786-O cells, as the gene encoding HIF1α is inherently truncated, and no functional HIF1α protein is present in this cell line [76].

To date, β-catenin is the only well-characterized transcription factor known to specially transactivate DCLK1-L, rather than DCLK1-S, by binding to the TCF/LEF sites within the DCLK1 α-promoter [31]. In this study, we identified β-catenin occupancy of the α-promoter by validated its selective transactivation of DCLK1-L over DCLK1-S in ccRCC. Subsequently, we confirmed that the hypoxia-PLOD2 axis governed the preferential activation of DCLK1-L in ccRCC by activating β-catenin, as shown by both loss- and gain-of-function investigations.

Recent advances in DCLK1 research underscore its isoform-specific functions in cancer [24, 25, 71, 77]. Sarkar et al. reported that DCLK1-S, rather than DCLK1-L, significantly increased the invasive potential of human colon cancer cells and that high DCLK1-S levels, but not DCLK1-L, were associated with poorer overall survival in colorectal cancer patients, underscoring the major oncogenic role of DCLK1-S in colon cancer [69]. Furthermore, Fan et al. identified DCLK1-S as an oncogenic isoform in human esophageal squamous cell carcinoma [25]. However, Qu et al. revealed that DCLK1-L (isoform 2) overexpression increased cell invasion and drug resistance in pancreatic cancer cells [77]. Additionally, Ge et al. showed that both DCLK1-L (isoform 1) and DCLK1-S (isoform 4) efficiently activated EMT and promoted tumor migration and invasion in pancreatic ductal adenocarcinoma [53]. Here, through CRISPR/Cas9-mediated specific depletion of DCLK1-L, we showed that DCLK1-L is essential for maintaining cancer stemness and EMT activation in ccRCC, as well as for hypoxia-induced malignant properties. Little is known about the functional differences between the two splice variants (isoform 1 and 2) of DCLK1-L. Here, we reveal that both DCLK1 isoforms 1 and 2 are significantly up-regulated in ccRCC and both can be stimulated by hypoxia and PLOD2. Detailed isoform-specific functional investigations demonstrated that both DCLK1-L variants trigger cancer stemness and EMT activation and mediate hypoxia-PLOD2-induced malignant properties in ccRCC.

Several studies have suggested DCLK1 as a druggable target against stemness, metastasis, and drug resistance in various tumors [30, 53, 57, 61, 77]; however, it remains unclear which patient population may benefit most from DCLK1-targeted intervention. Given that our data identify a hypoxia-HIF2α-PLOD2 axis driving DCLK1-L activation in ccRCC, we speculated that hypoxic ccRCC patients with high PLOD2 expression may derive therapeutic benefits from DCLK1-targeted intervention. Indeed, pharmacological targeting of DCLK1 using the specific inhibitor DCLK1-IN-1 abrogated hypoxia- and PLOD2-induced stemness and EMT activation in ccRCC cell lines in vitro and suppressed growth, EMT, and stemness properties of PLOD2-rich ccRCC xenografts in vivo. Consistently, the therapeutic value of a DCLK1 inhibitor against ccRCC invasion and stemness was also shown in a recent in vitro study [30].

Collectively, our data reveal a shift in *DCLK1* AP model choice during ccRCC tumorigenesis, emphasize the tumorigenic contribution of DCLK1-L variants in ccRCC stemness and aggressiveness, identify a hypoxia-HIF2α-PLOD2 axis that activates β-catenin to preferentially drive α-promoter activation and DCLK1-L variants production, and propose DCLK1-L as a potential therapeutic target in hypoxic, PLOD2-rich ccRCCs. Our study also has several limitations, including reliance on a single data set (TCGA), the use of specific cell lines, and the small sample sizes in both in vitro and in vivo studies.

## Supplementary information

Supplementary materials

Raw data -Western blot

## Data Availability

The datasets used or analyzed in this study are available from the corresponding author on reasonable request.
