# Supplementary material for: Hypoxic stimulation of DCLK1 transcription and alternative-promoter switching fuels tumor malignancy in clear cell renal cell carcinoma
Source: Cell Death Dis. 2025 Aug 7;16(1):594. doi: 10.1038/s41419-025-07916-2 (PMC12332081; doi:10.1038/s41419-025-07916-2)
Supplement: Supplementary file 1 — Supplementary materials [file 41419_2025_7916_MOESM1_ESM.pdf]

Supplementary Materials (Figure S1-S7; Table S1-S2)

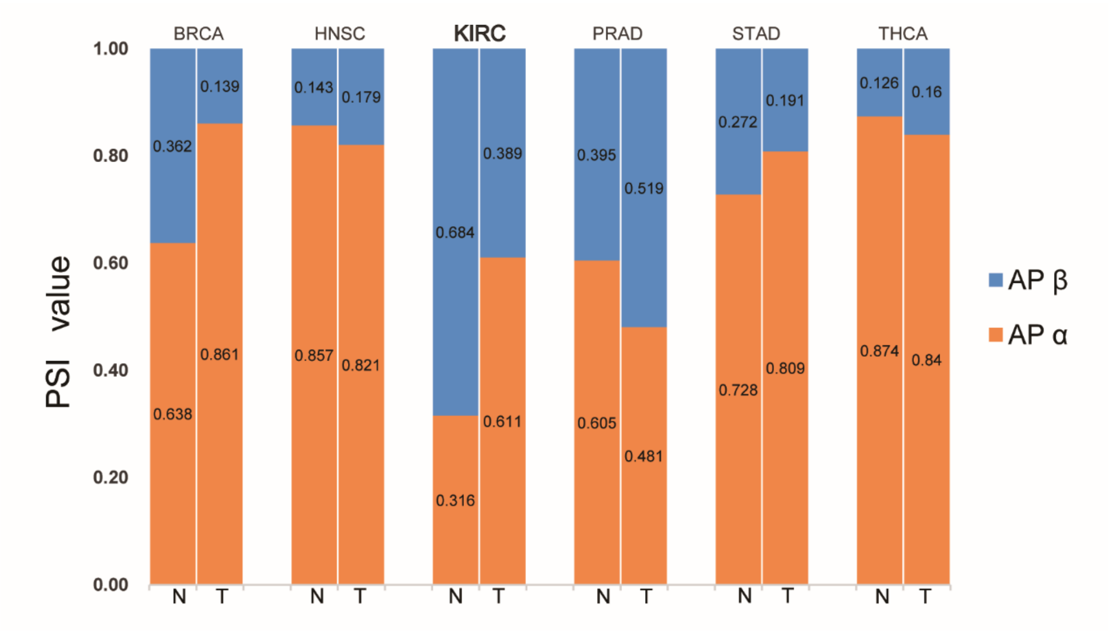

Figure S1. PSI values for tumor and normal samples in the TCGA SpliceSeq database.

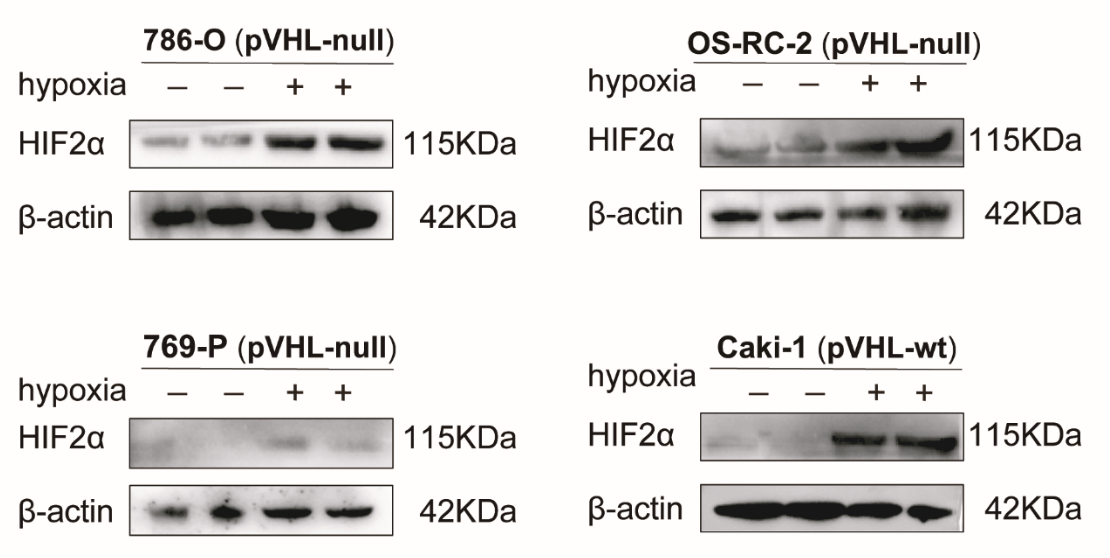

Figure S2. Hypoxia induced HIF2α activation in ccRCC cell lines with and without *VHL* mutation. Western blotting examination of the effect of hypoxia on HIF2α expression at the protein level in 786-O, OS-RC-2, 769-P and Caki-1 cells.

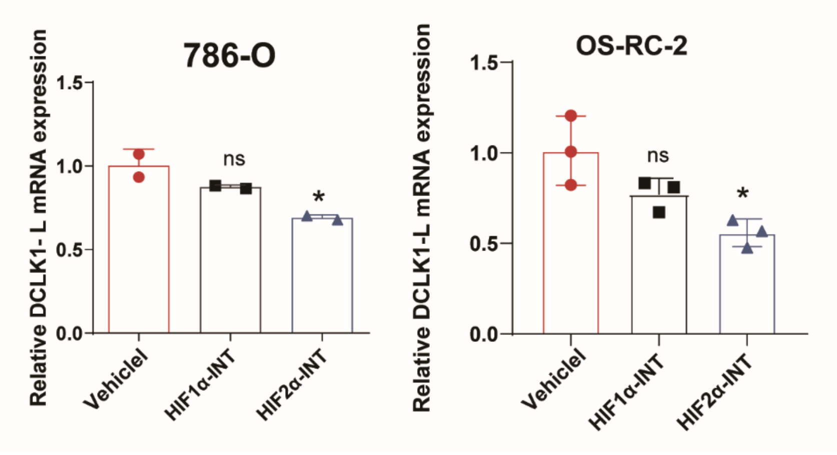

Figure S3. RT-qPCR assays to examine the effect of HIF-1α-INT and HIF-2α-INT treatment on the expression of DCLK1-L at the mRNA level. HIF1α-INT: the HIF-1α-Inhibitor BAY87-2243; HIF2α-INT: the HIF-2α-Inhibitor PT2385.

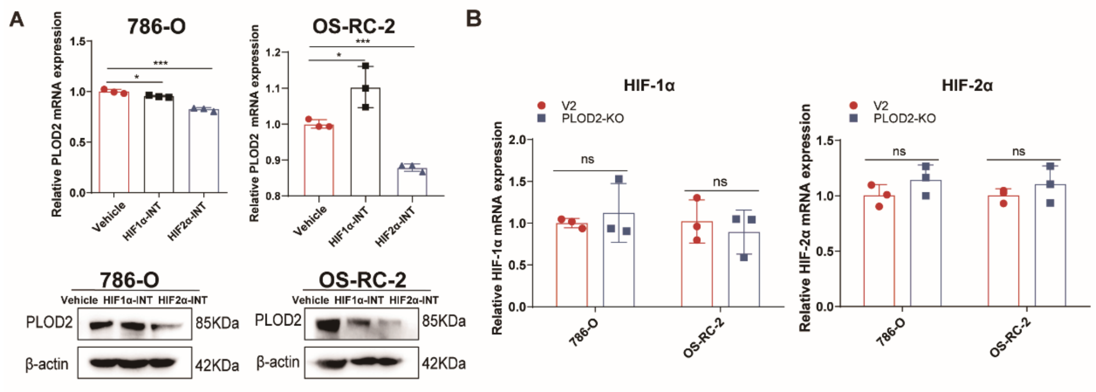

Figure S4. HIF2α acts upstream of PLOD2. (**A**) RT-qPCR and Western blotting assays to detect the effects of HIF1α-INT and HIF2α-INT treatment on the expression of PLOD2 in 786-O and OS-RC-2 cells. (**B**) RT-qPCR assays to examine the effect of PLOD2 ablation on the expression of HIF1α and HIF2α in 786-O and OS-RC-2 cells. The data are presented as the mean ± SD from three independent experiments performed in triplicate. **p* < 0.05, ****p* < 0.001; ns, non-significant.

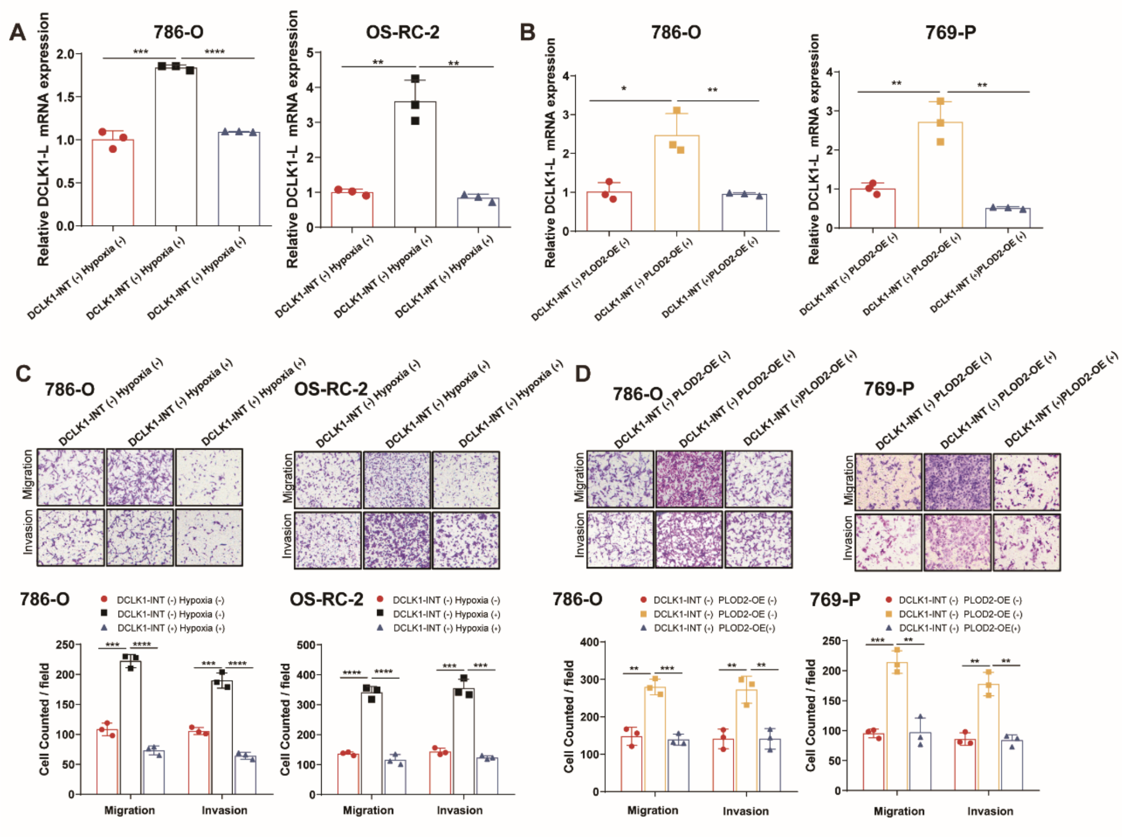

Figure S5. DCLK-IN-1 abrogated hypoxia- and PLOD2- induced ccRCC invasiveness. (**A**) RT-qPCR validation of the effect of DCLK1 inhibitor (DCLK1-INT) on reversing hypoxia-activated DCLK1-L expression at the mRNA level in 786-O and OS-RC-2 cells. (**B**) RT-qPCR determination of the effect of DCLK1-INT on reversing PLOD2 overexpression-activated DCLK1-L expression at the mRNA level in 786-O and OS-RC-2 cells. (**C**) Transwell assays to examine the effect of DCLK1-INT on hypoxia-driven cell invasion and migration in 786-O and OS-RC-2 cells. (**D**) Transwell assays to examine the effect of DCLK1-INT on PLOD2 overexpression-driven cell invasion and migration in 786-O and OS-RC-2 cells. The data are presented as the mean ± SD from three independent experiments performed in triplicate. *$p < 0.05$, **$p < 0.01$, ***$p < 0.001$, ****$p < 0.0001$.

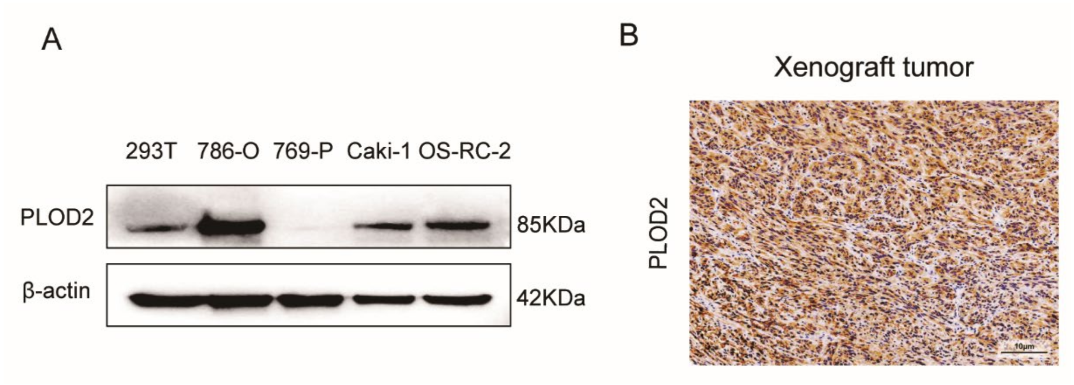

Figure S6. Abundant PLOD2 expression in 786-O cells. (**A**) Western blotting examination of PLOD2 in different cell lines. (**B**) IHC examination of PLOD2 in 786-O-derived subcutaneous xenograft.

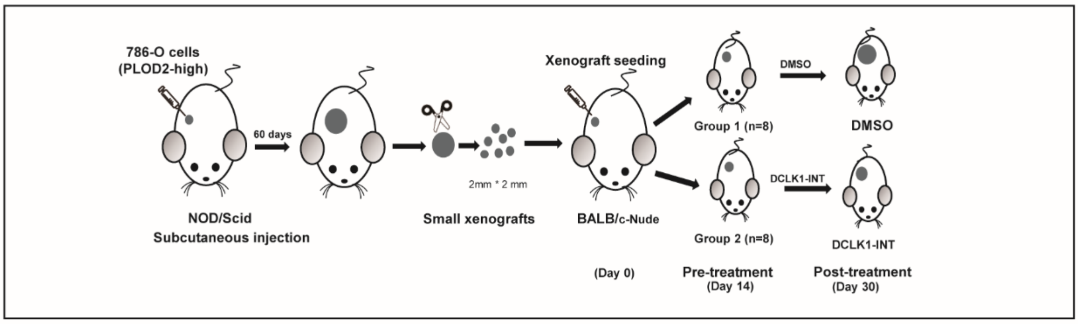

Figure S7. The flow diagram for the *in vivo* mouse experiments.

Supplementary Table 1. Antibodies used in this study.

Antibody (catalog number)	Concentration	Company
DCLK1-isoform1(D203L)	1:1000	Cell Signaling Technology
DCLK1-isoform2/4(ab31704)	1:1000	Abcam
PLOD2(21214-1-AP)	1:1000	Proteintech
ZO1(AF0321)	1:1000	Beyotime
ZEB1(D80D3)	1:1000	Cell Signaling Technology
E-Cadherin(24E10)	1:1000	Cell Signaling Technology
N-Cadherin(D4R1H)	1:1000	Cell Signaling Technology
Vimentin(10366-1-AP)	1:1000	Proteintech
Slug(C19G7)	1:1000	Cell Signaling Technology
Snail(C15D3)	1:1000	Cell Signaling Technology
CD44(156-3C11)	1:1000	Cell Signaling Technology
Sox2(11064-1-AP)	1:1000	Proteintech
Bmi-1(D20B7)	1:1000	Cell Signaling Technology
Oct4(11263)	1:1000	Proteintech
Nanog(14295-1-AP)	1:1000	Proteintech
β-actin(66009-1-Ig)	1:1000	Proteintech
β-catenin(D10A8)	1:1000	Cell Signaling Technology
HRP-labeled Goat Anti-Rabbit IgG (H+L) (A0208)	1:8000	Beyotime
HRP-labeled Goat Anti-Mouse IgG (H+L) (A0216)	1:8000	Beyotime

Supplementary Table 2. Primers used in this study.

Primers names	Assay	Primers sequences (5’→3’)
Human-DCLK1-L-Forward	RT-qPCR	CGGTCCACATGCAATAAAAA
Human-DCLK1-L-Reverse	RT-qPCR	GATATCACCGATGCCATCAAG
Human-DCLK1-S-Forward	RT-qPCR	ACACTAAGACTGTGTCCATGTTAGAACTC
Human-DCLK1-S-Reverse	RT-qPCR	AAGCCTTCCTCCGACACTTCT
Human-PLOD2-Forward	RT-qPCR	CATGGACACAGGATAATGGCTG
Human-PLOD2-Reverse	RT-qPCR	AGGGGTTGGTTGCTCAATAAAAA
Human-CD44-Forward	RT-qPCR	CTGCCGCTTTGCAGGTGTA
Human-CD44-Reverse	RT-qPCR	CATTGTGGGCAAGGTGCTATT
Human-Sox2-Forward	RT-qPCR	CATCACCCACAGCAAATGAC
Human-Sox2- Reverse	RT-qPCR	TTTTTCGTCGCTTGGAGACT
Human-Oct4-Forward	RT-qPCR	ACATCAAAGCTCTGCAGCAAAGAACT
Human-Oct4- Reverse	RT-qPCR	CTGAATACCTTCCCAAATAGAACCC
Human-Nanog-Forward	RT-qPCR	ACATGCAACCTGAAGACGTGTG
Human-Nanog-Reverse	RT-qPCR	CATGGAAACCAGAACACGTGG
Human-Bmi-1-Forward	RT-qPCR	TCATGGTCATCCTTCTGCTGATGCTG
Human-Bmi-1-Reverse	RT-qPCR	GCATGAGCATCACAGTCATTGCTGCT
Human-E-cadherin-Forward	RT-qPCR	GTCACTGACACCAACGATAATCCT
Human-E-cadherin-Reverse	RT-qPCR	TTTCAGTGTGGTGATTACGACGTTA
Human-N-cadherin-Forward	RT-qPCR	TCAGGCGTCTGTAGAGGCTT
Human-N-cadherin-Reverse	RT-qPCR	ATGCACATCCTTCGATAAGACTG
Human-ZO1-Forward	RT-qPCR	CAACATACAGTGACGCTTCACA
Human-ZO1-Reverse	RT-qPCR	CACTATTGACGTTTCCCCACTC
Human-ZEB1-Forward	RT-qPCR	GATGATGAATGCGAGTCAGATGC
Human-ZEB1-Reverse	RT-qPCR	ACAGCAGTGTCTTGTTGTTGT
Human-Vimentin-Forward	RT-qPCR	CCTGAACCTGAGGGAAACTAA
Human-Vimentin-Reverse	RT-qPCR	GCAGAAAGGCACTTGAAAGC-
Human-Snail-Forward	RT-qPCR	GGCAGGGGCAGGTATGGAGA
Human-Snail-Reverse	RT-qPCR	GCCATGTCCGGACCCACACTG-
Human-Slug-Forward	RT-qPCR	TGTGACAAGGAATATGTGAGCC
Human-Slug-Reverse	RT-qPCR	TGAGCCCTCAGATTTGACCTG
Human-β-actin-Forward	RT-qPCR	CATGTACGTTGCTATCCAGGC
Human-β-actin-Reverse	RT-qPCR	CTCCTTAATGTCACGCACGAT
Human-β-catenin-Forward	RT-qPCR	AGCTTCCAGACACGCTATCAT
Human-β-catenin- Reverse	RT-qPCR	CGGTACAACGAGCTGTTTCTAC
Human-DCLK1-α-promoter -Forward-1	ChIP-qPCR	AGAGCTGTGTCTGCTTGG
Human-DCLK1-α-promoter -Reverse-1	ChIP-qPCR	GTTCATTCCAGGGCAGCTTA
Human-DCLK1-α-promoter -Forward-2	ChIP-qPCR	TAAGCTGCCCTGGAATGAAC
Human-DCLK1-α-promoter -Reverse-2	ChIP-qPCR	CCCAAGCTATGCACTCTGGT
