# Supplementary material for: Hypoxic stimulation of DCLK1 transcription and alternative-promoter switching fuels tumor malignancy in clear cell renal cell carcinoma
Source: Cell Death Dis. 2025 Aug 7;16(1):594. doi: 10.1038/s41419-025-07916-2 (PMC12332081; doi:10.1038/s41419-025-07916-2)

Figure 1E- raw data

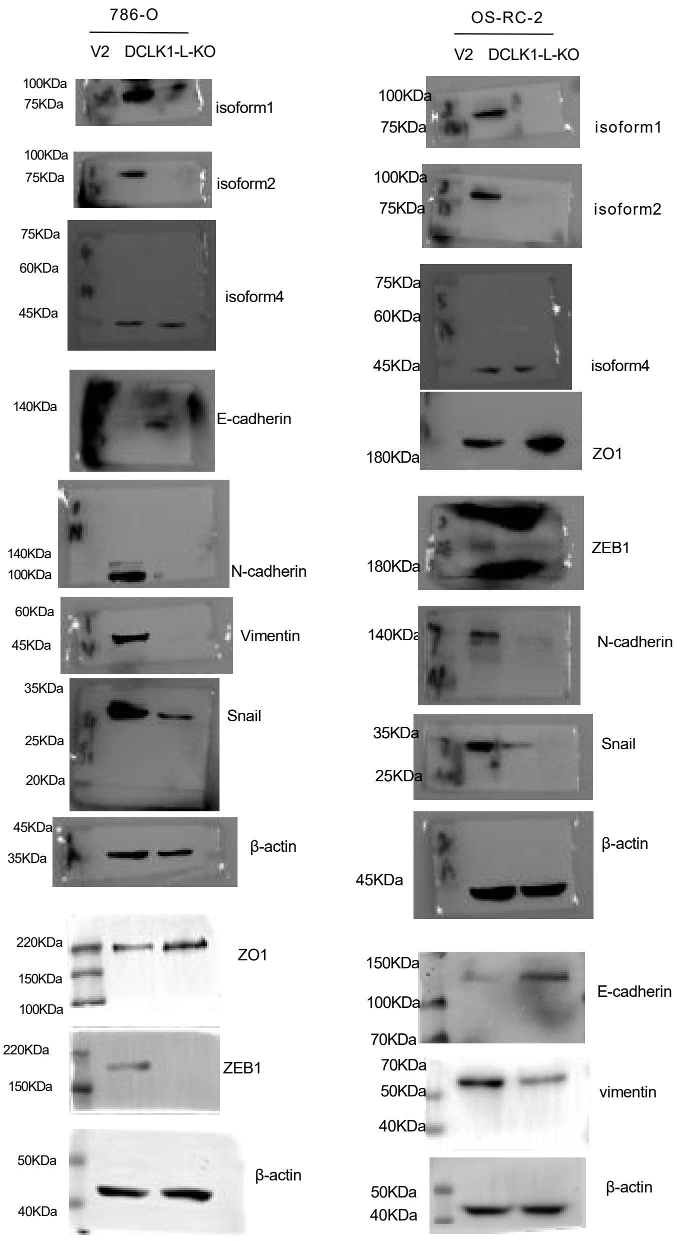

Figure 1H-raw data

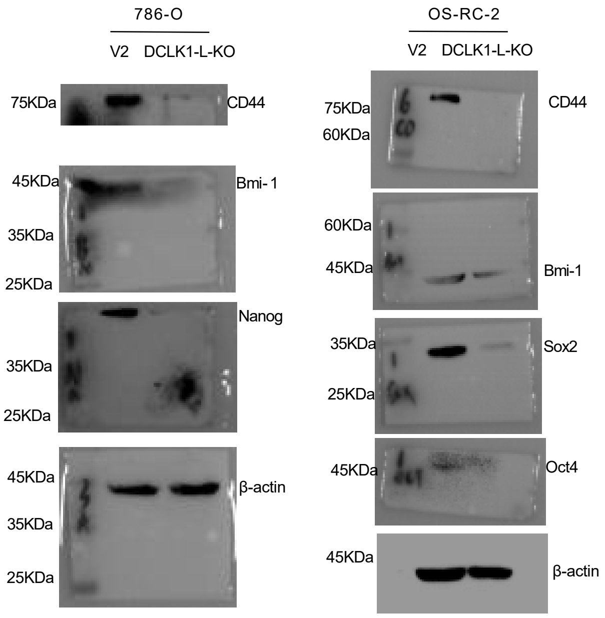

Figure 2B-raw data

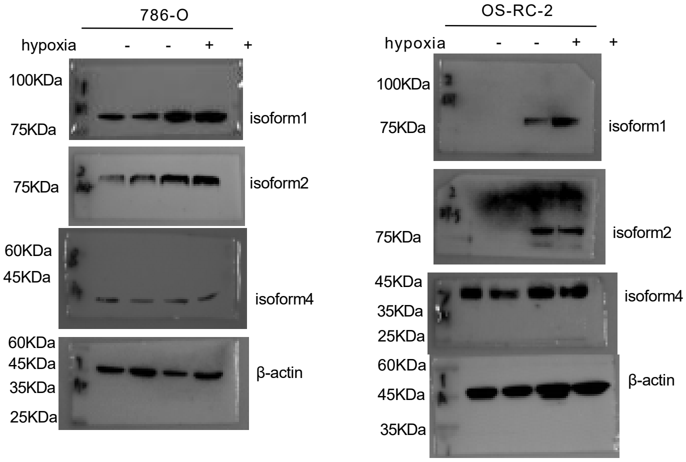

Figure 2D-raw data

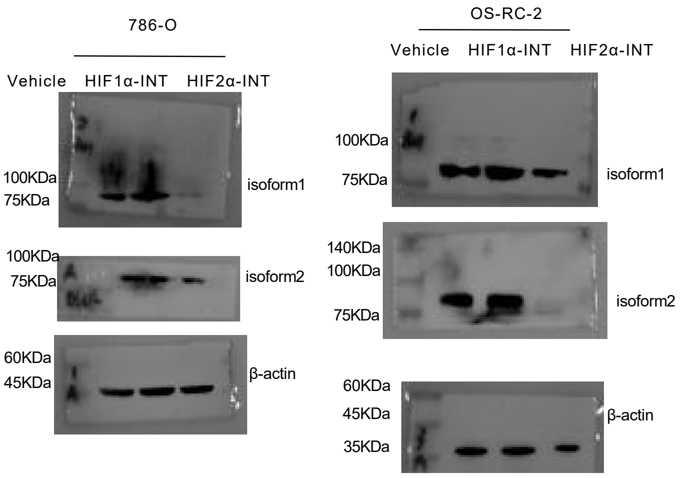

Figure 2F-raw data

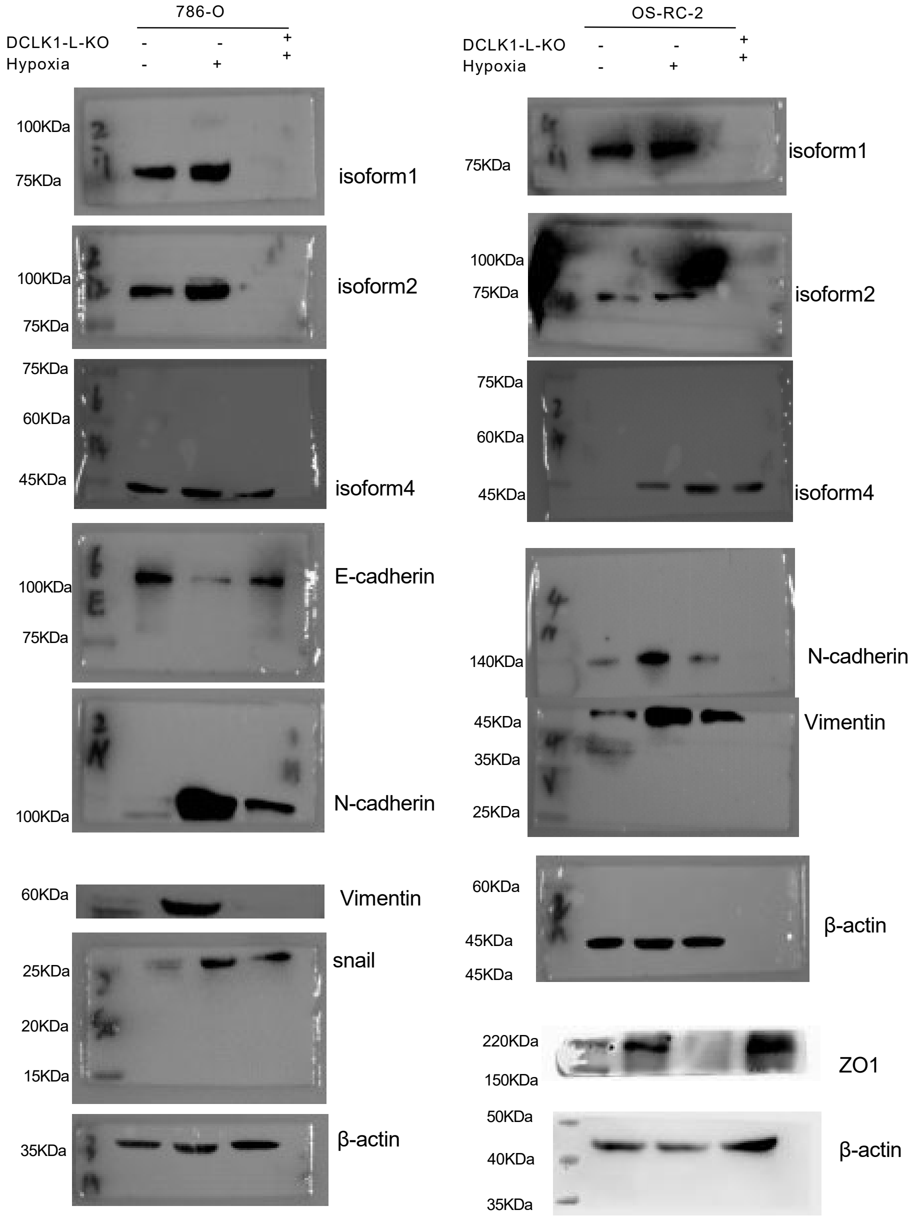

Figure 2I-raw data

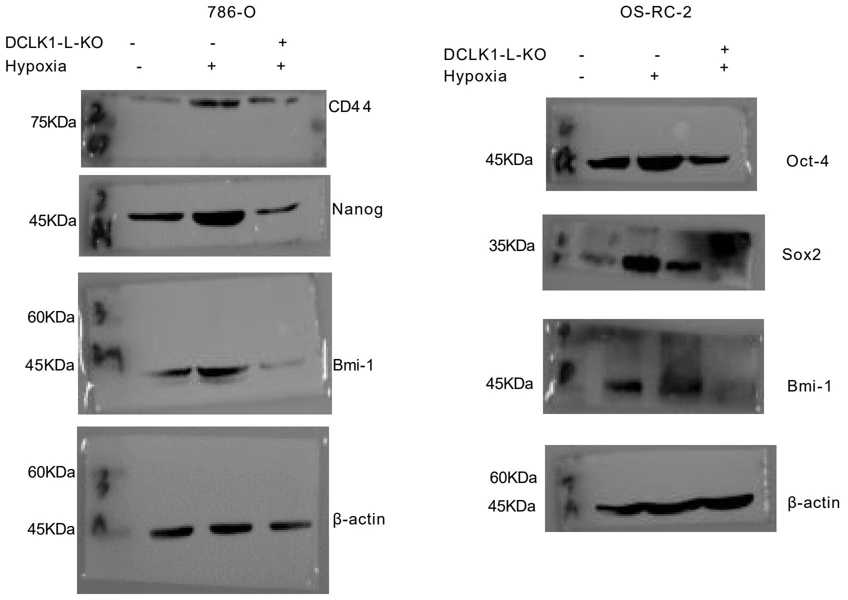

Figure 3B-raw data

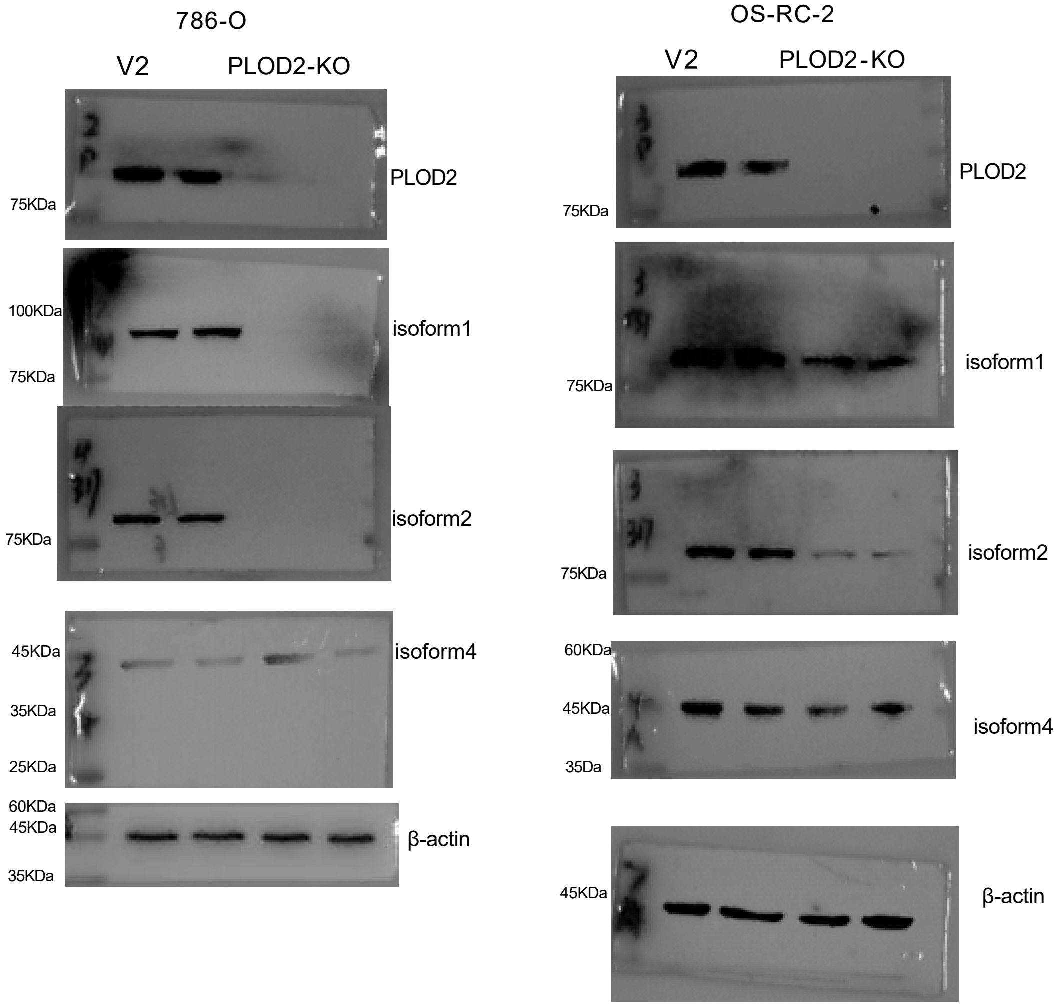

Figure 3F-raw data

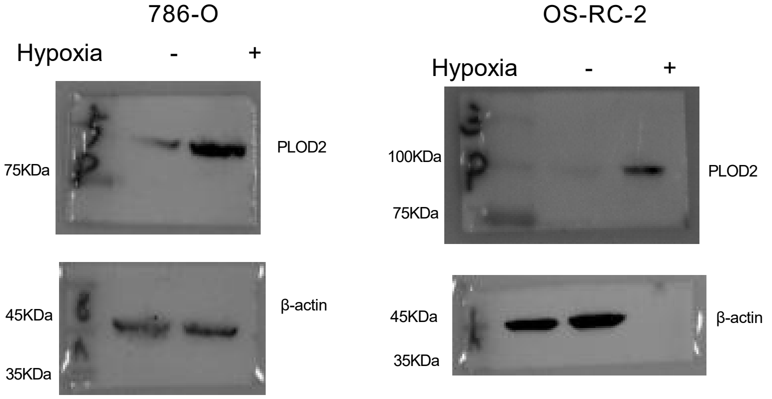

Figure 3H-raw data

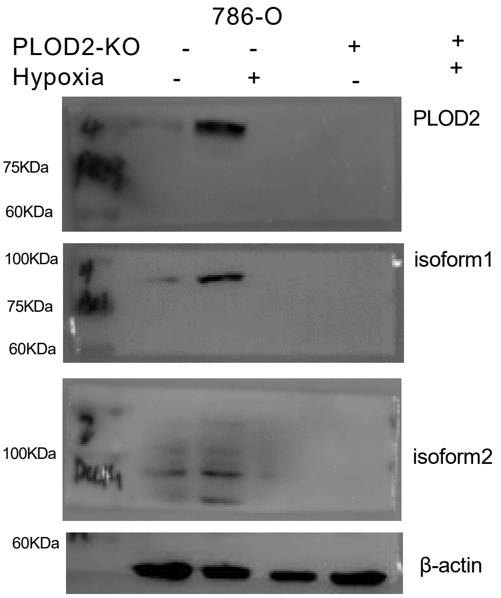

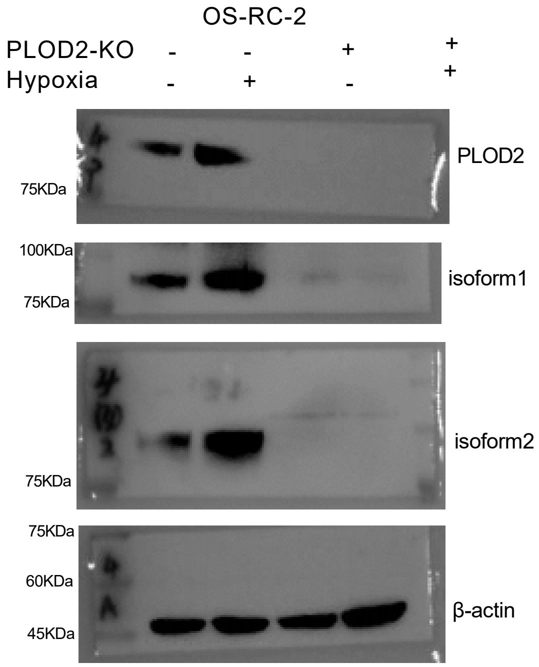

Figure 4C-raw data

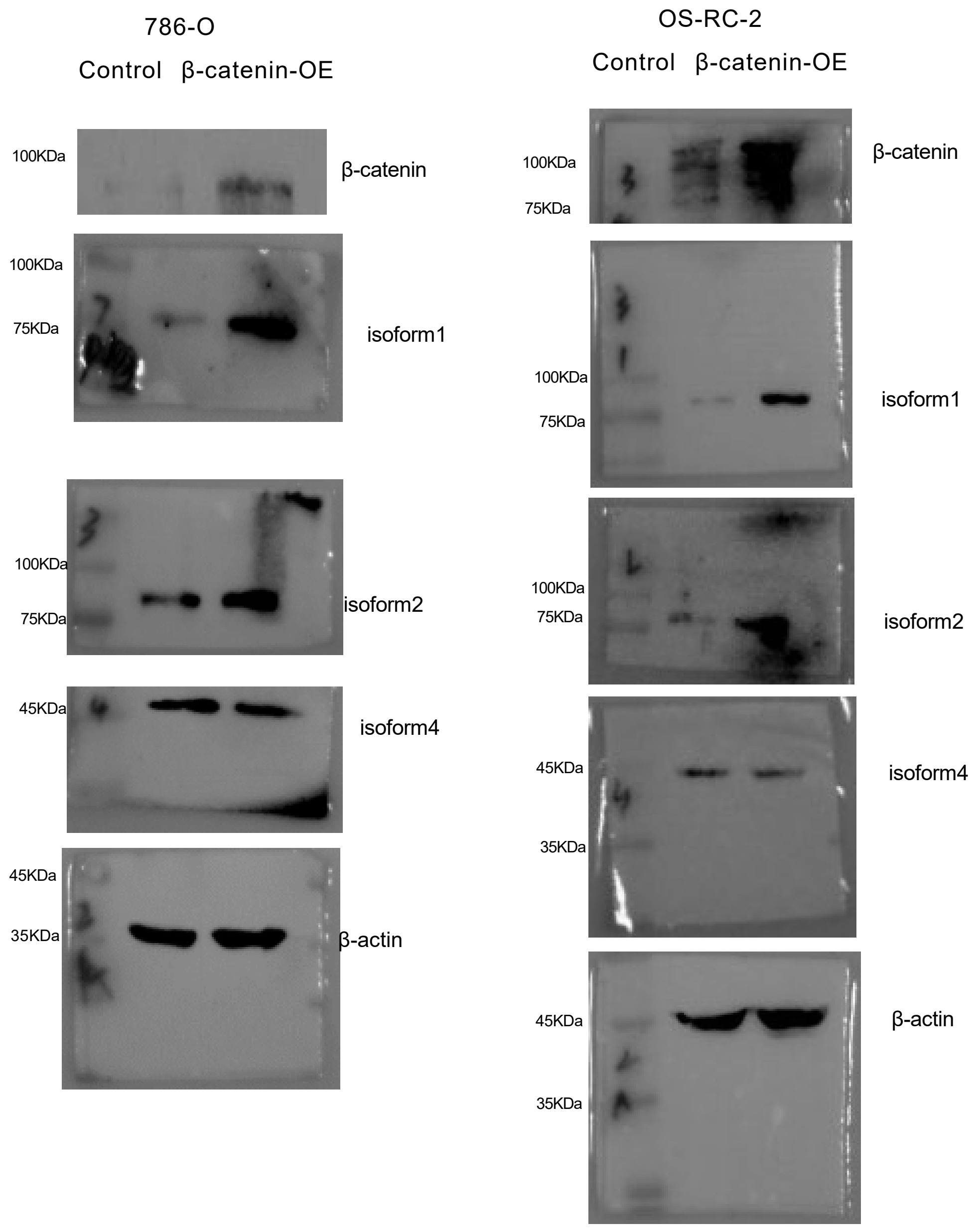

Figure 4D-raw data

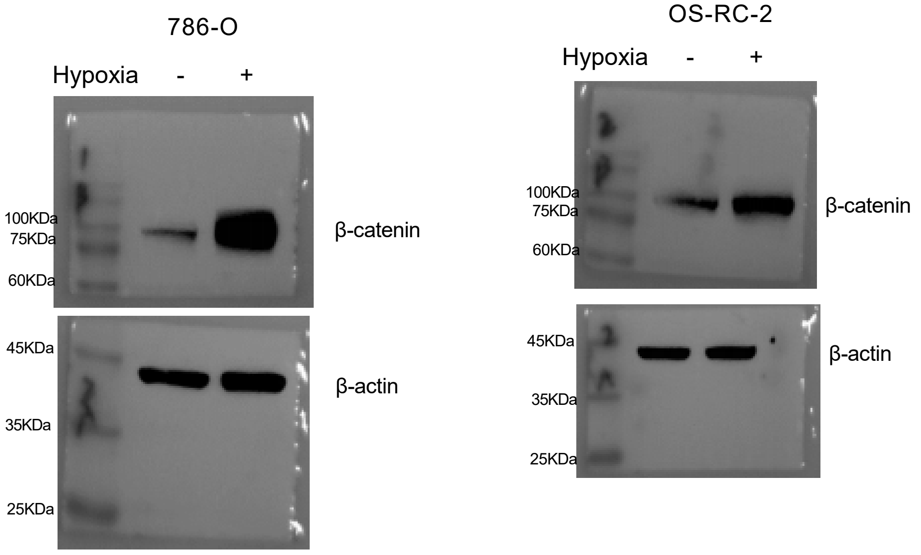

Figure 4E-raw data

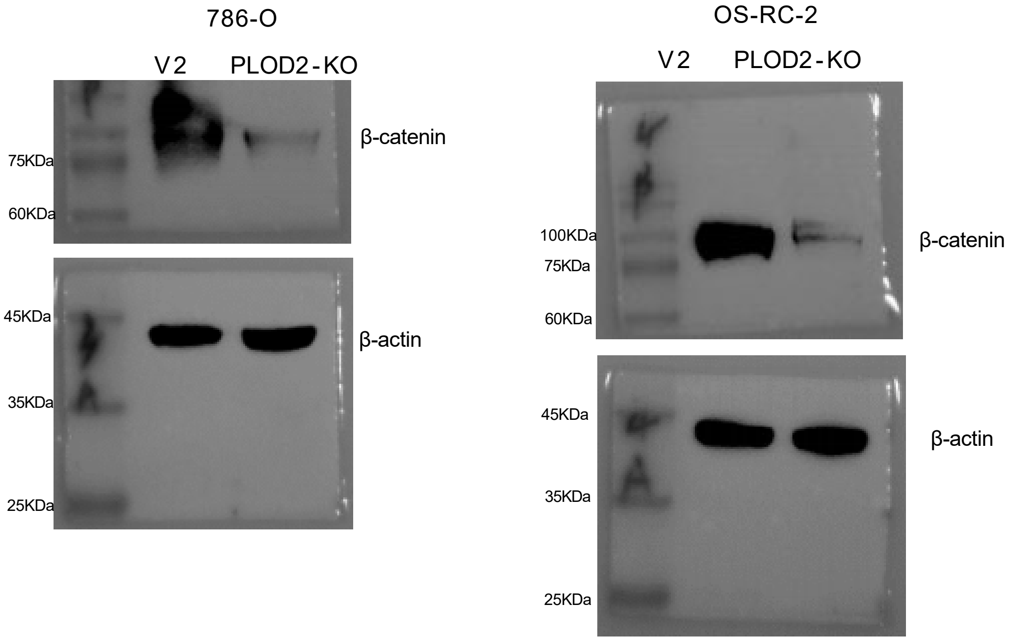

Figure 4G-raw data

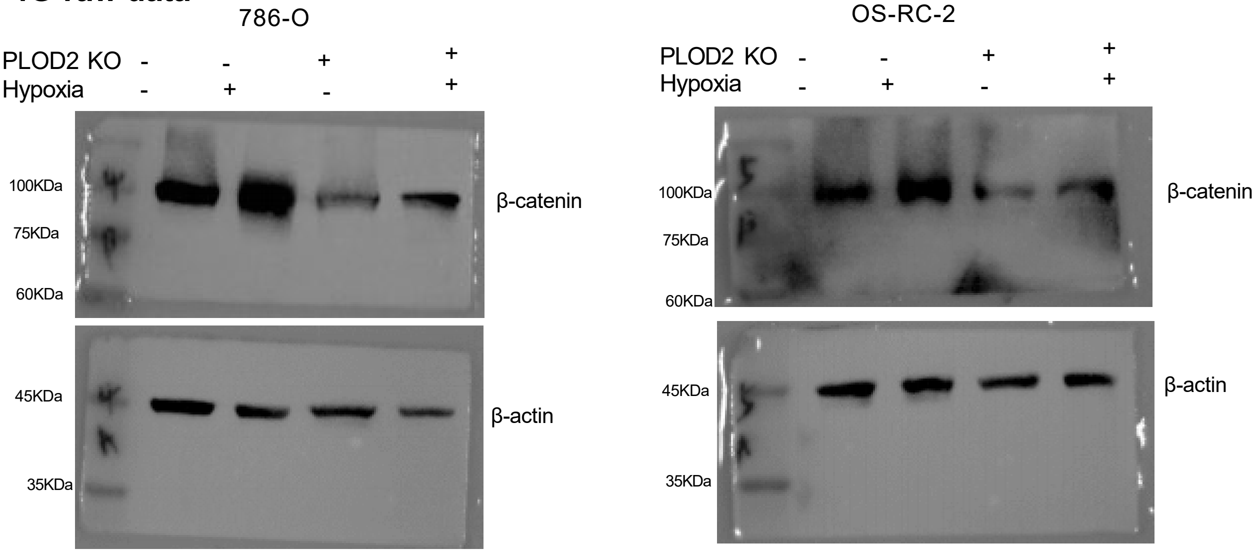

Figure 4I-raw data

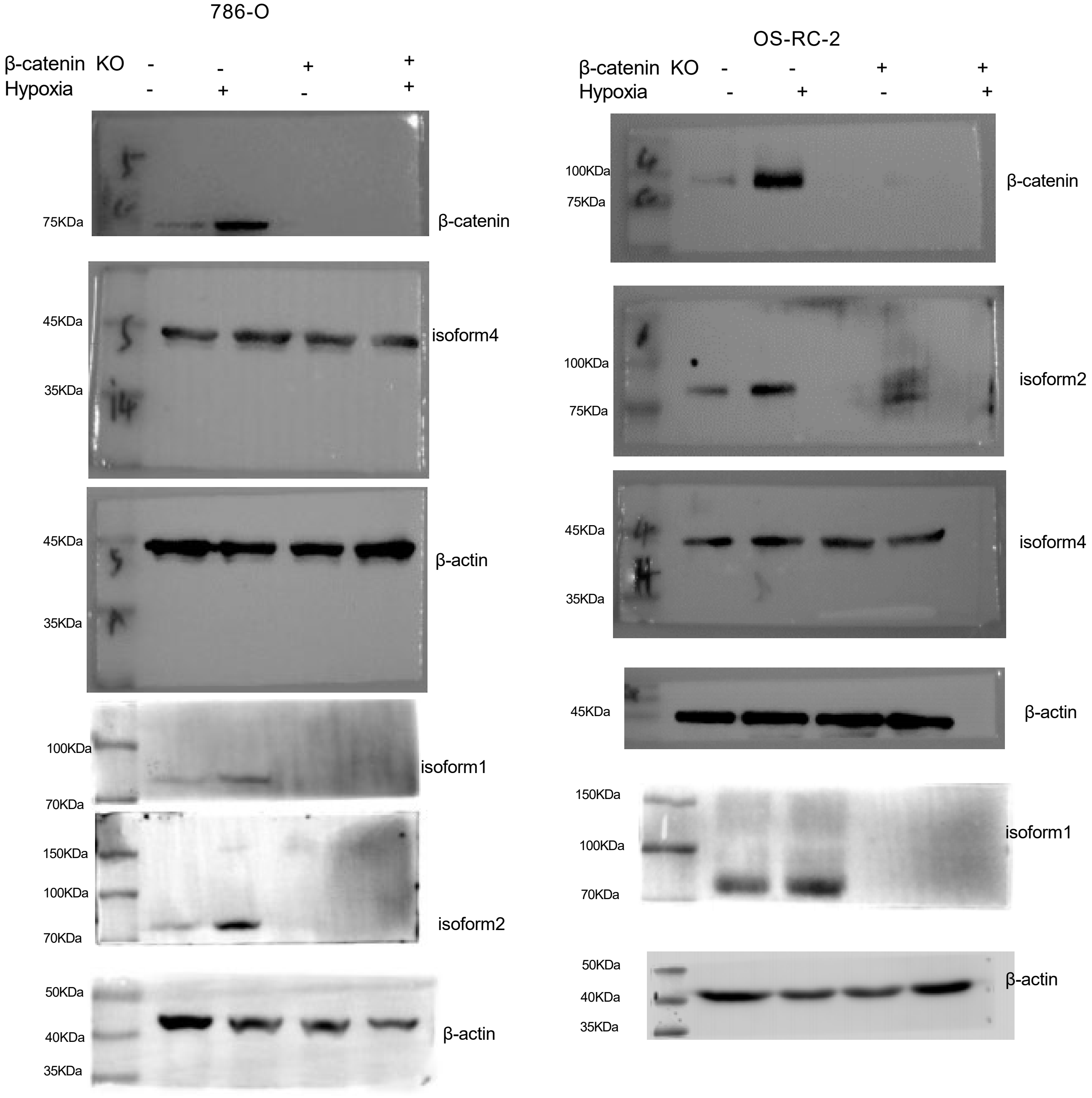

Figure 4K-raw data

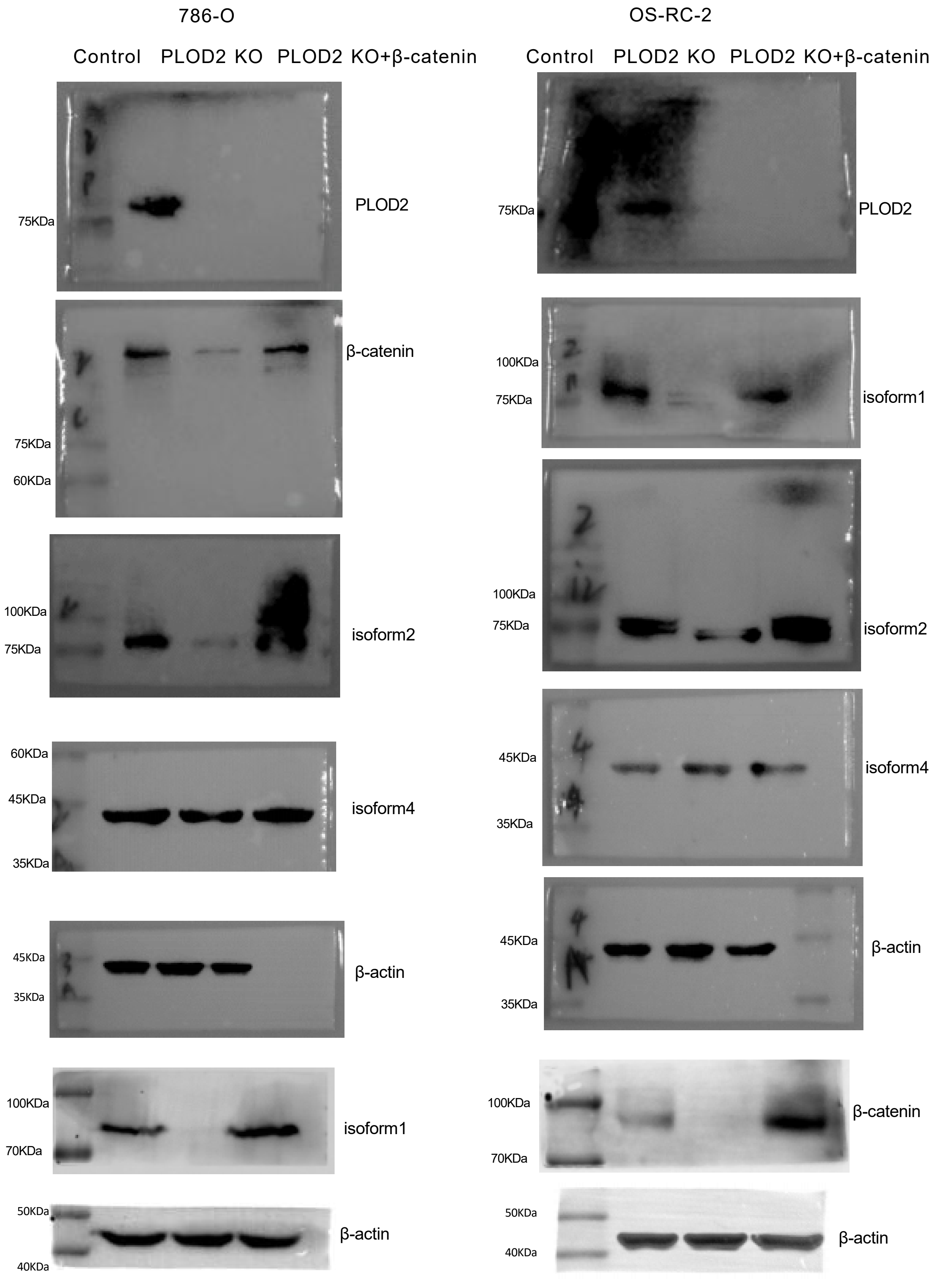

Figure 5B-raw data

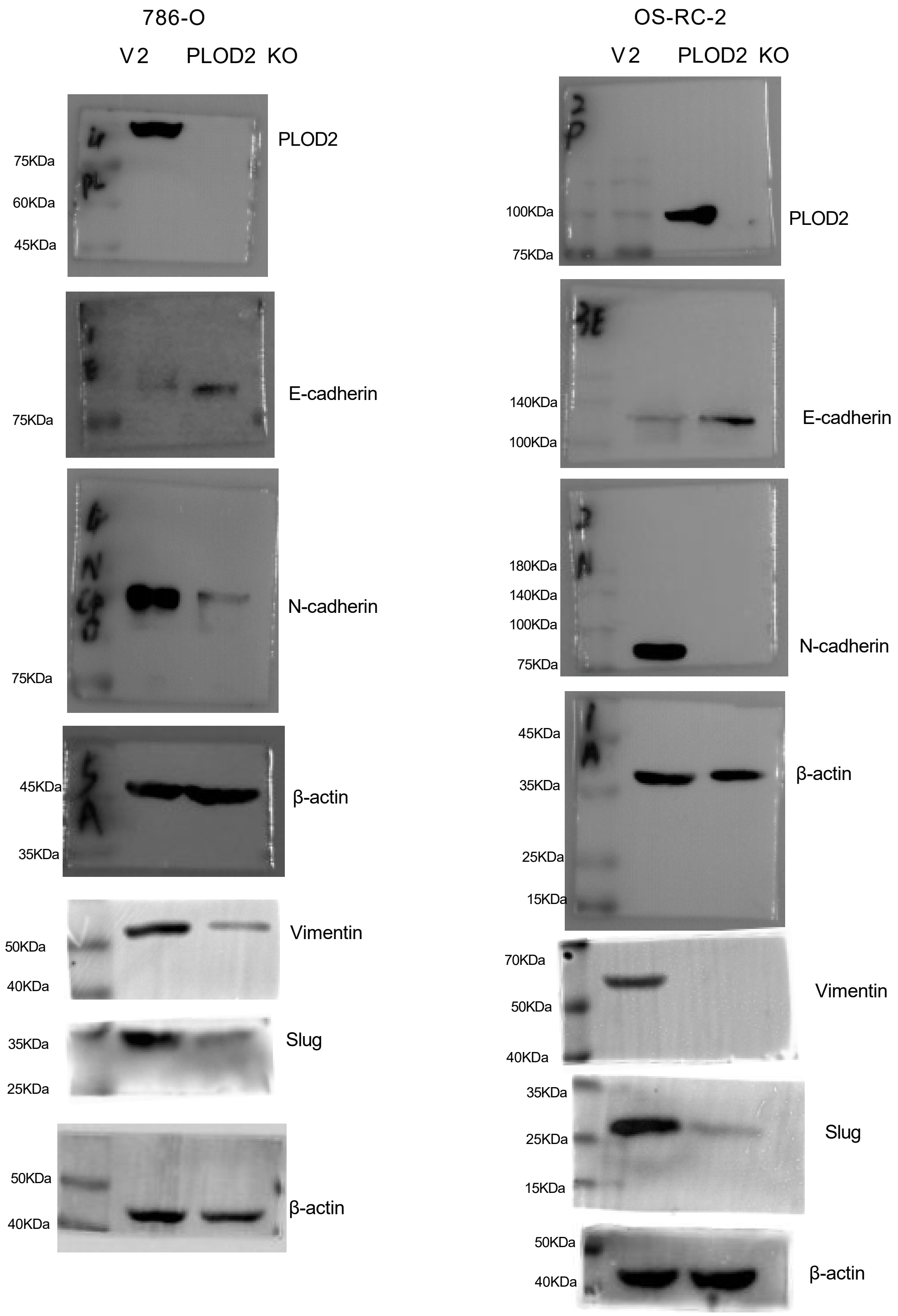

Figure 5D-raw data

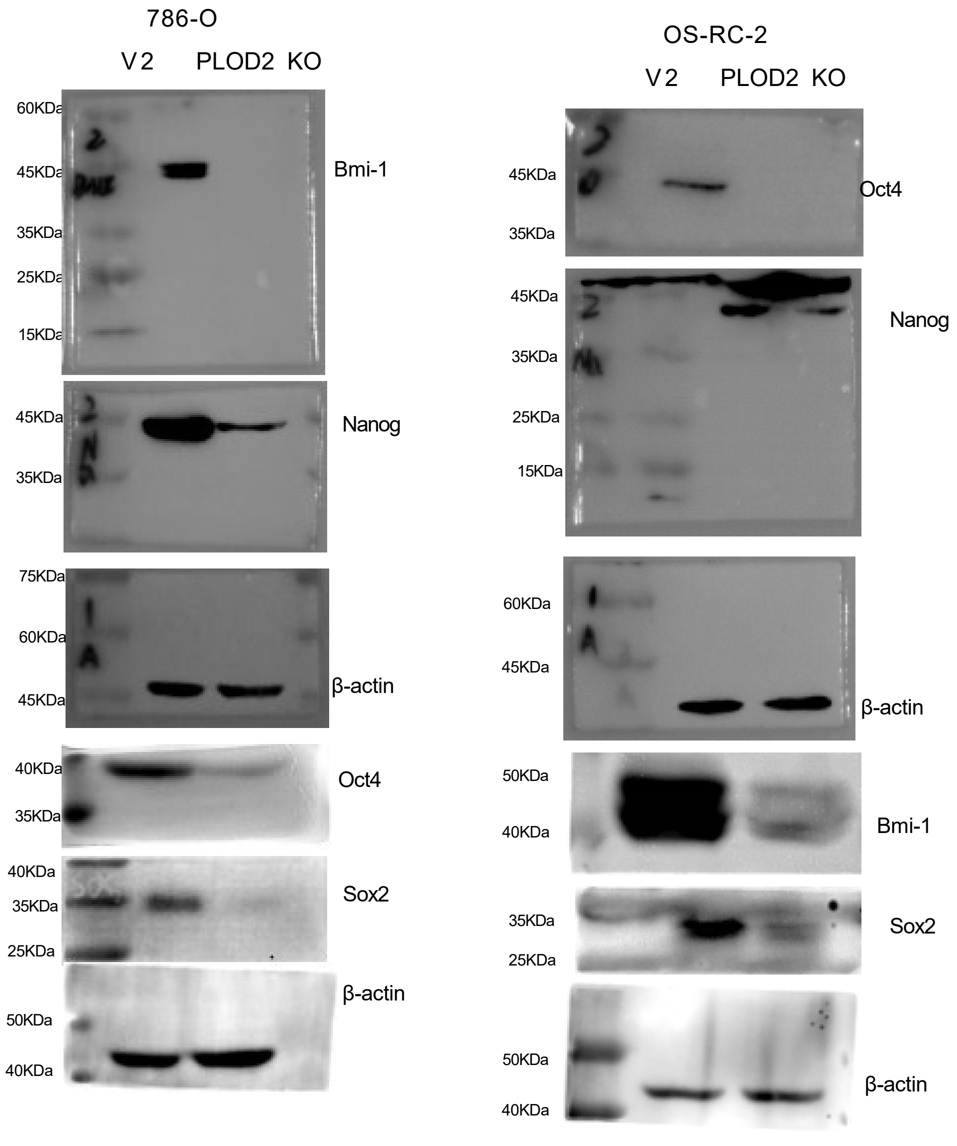

Figure 5F-raw data

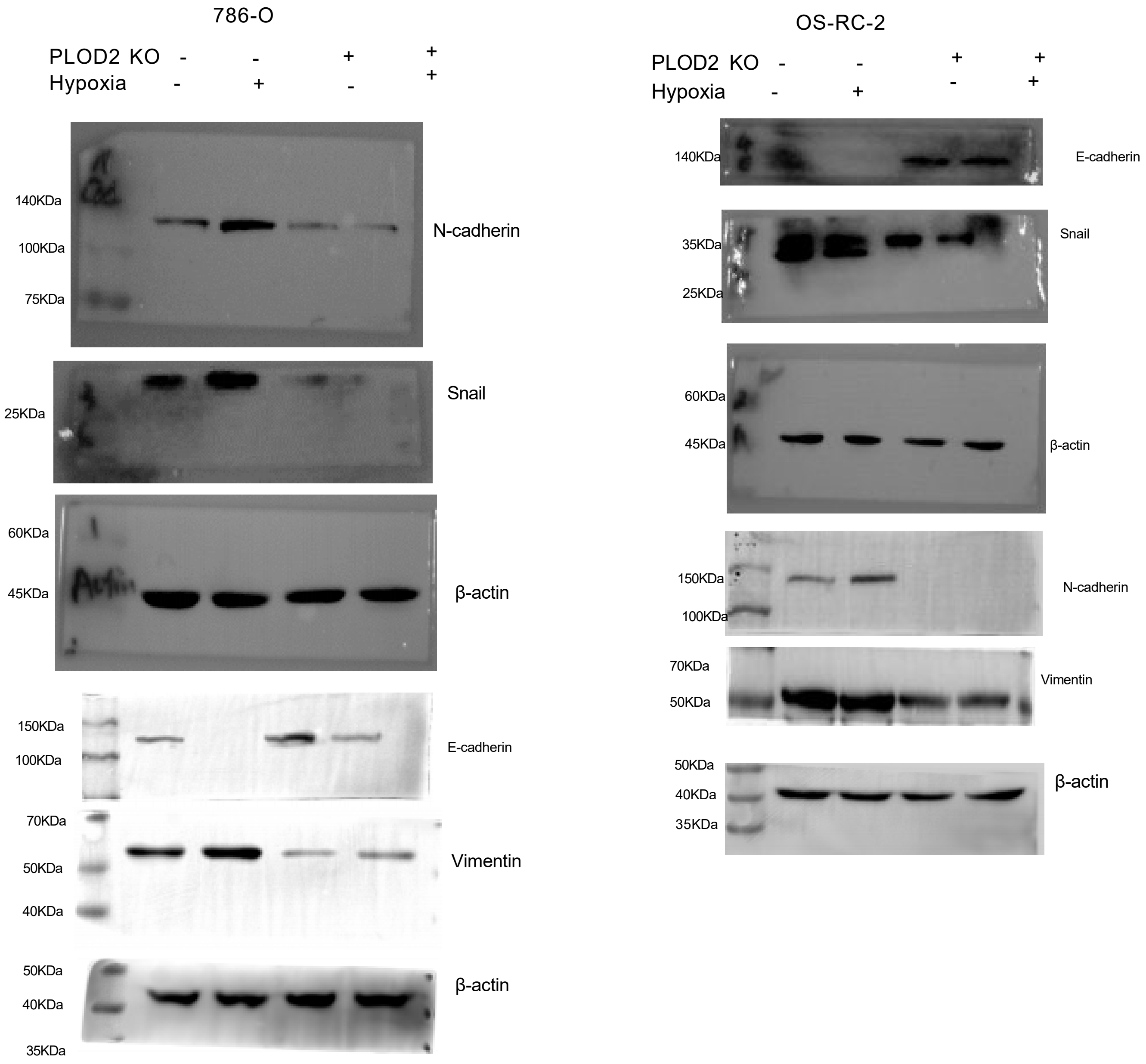

Figure 5I-raw data

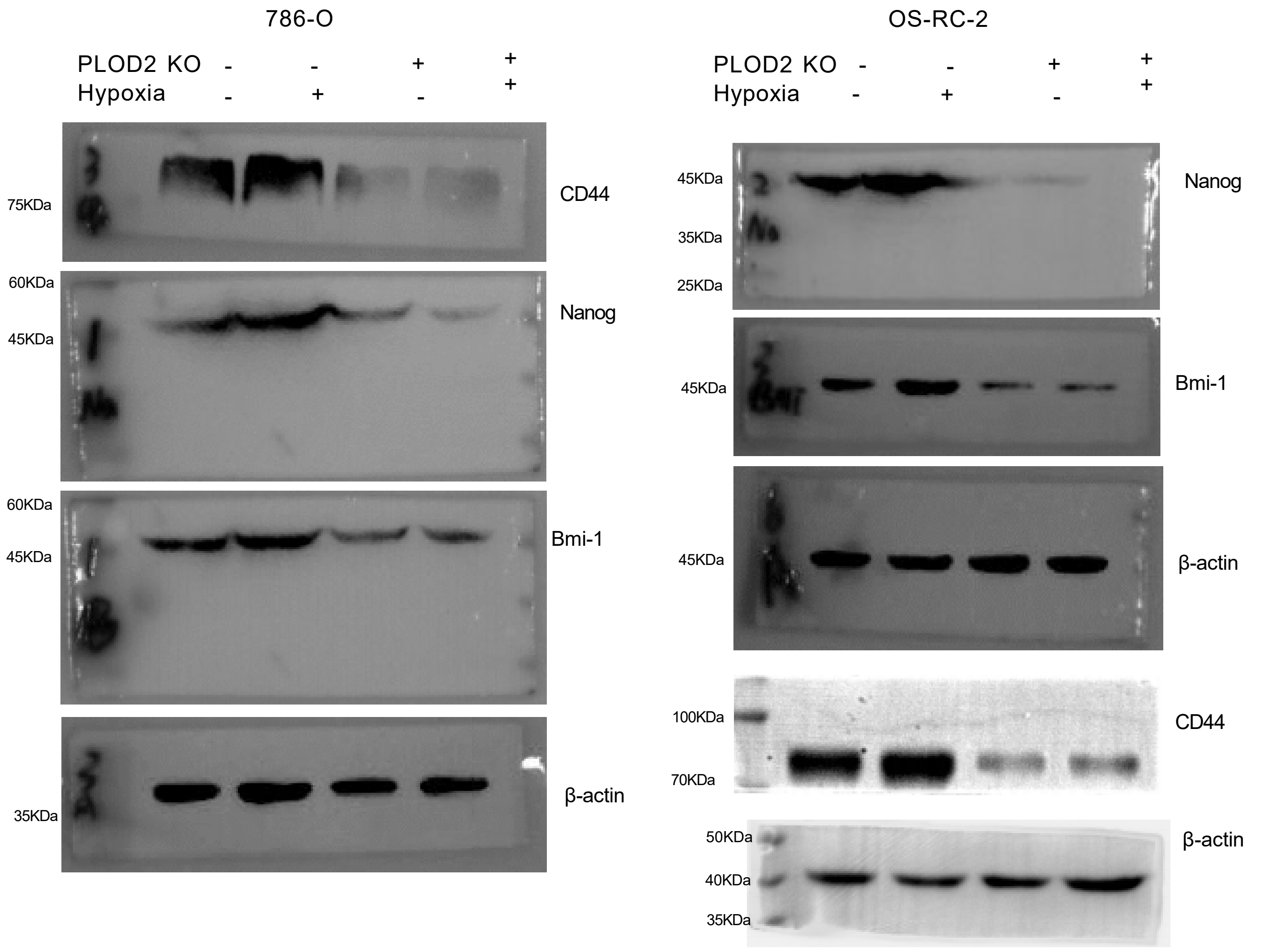

Figure 6A-raw data

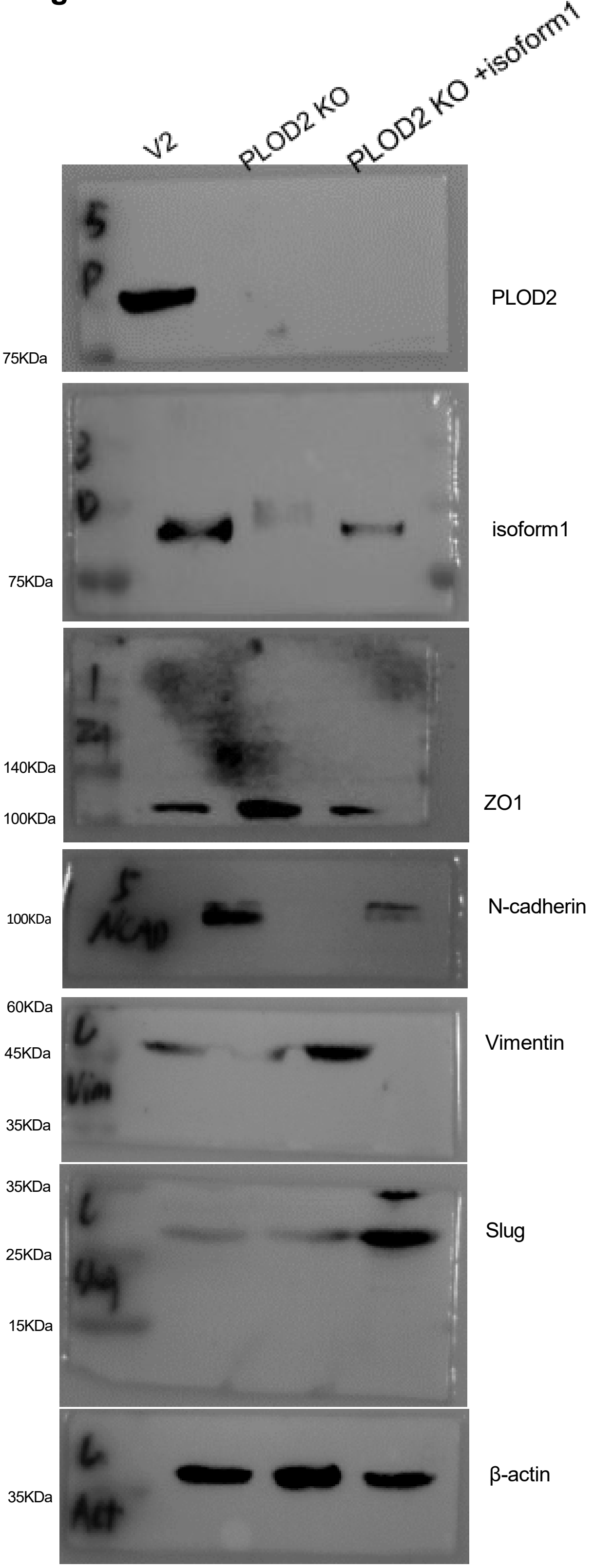

Figure 6B-raw data

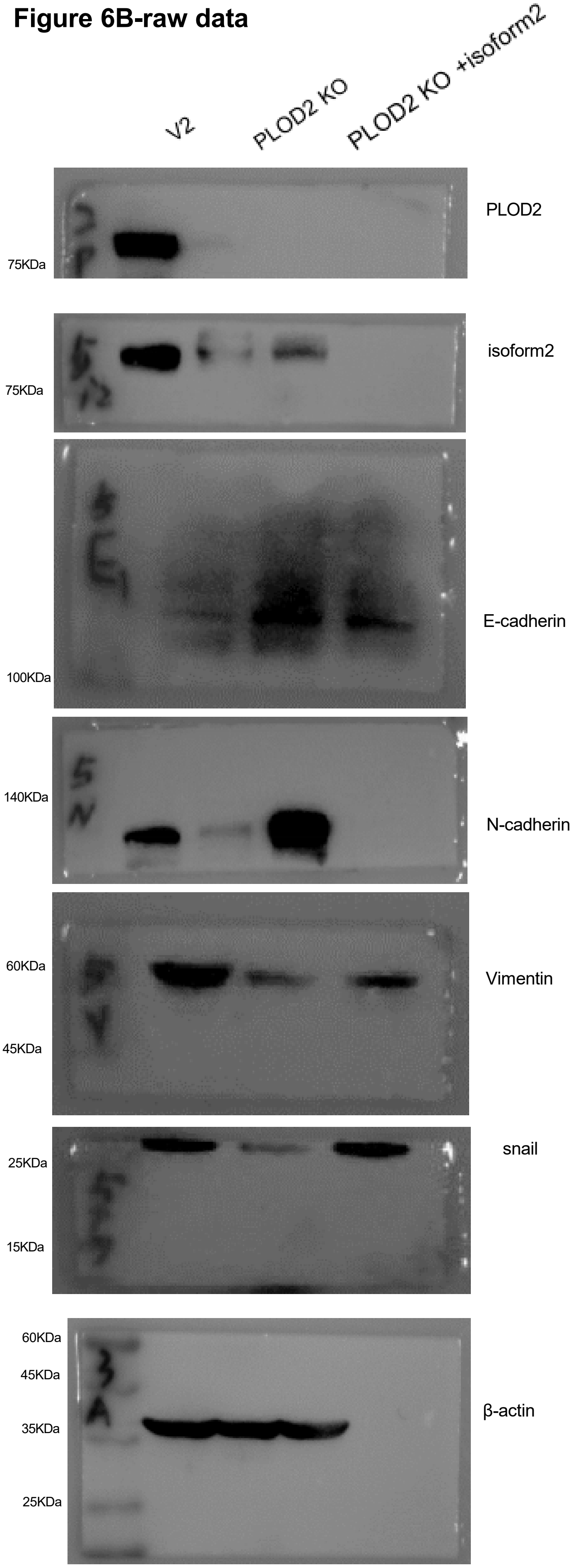

Figure 6E-raw data

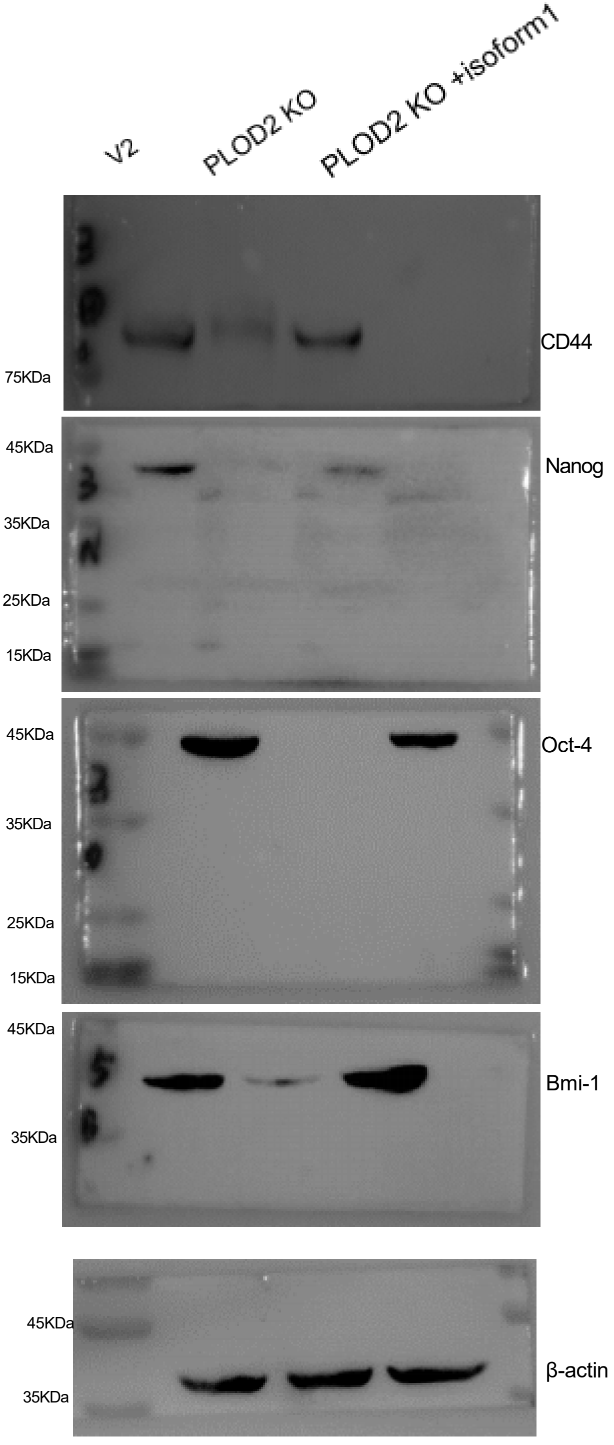

Figure 6F-raw data

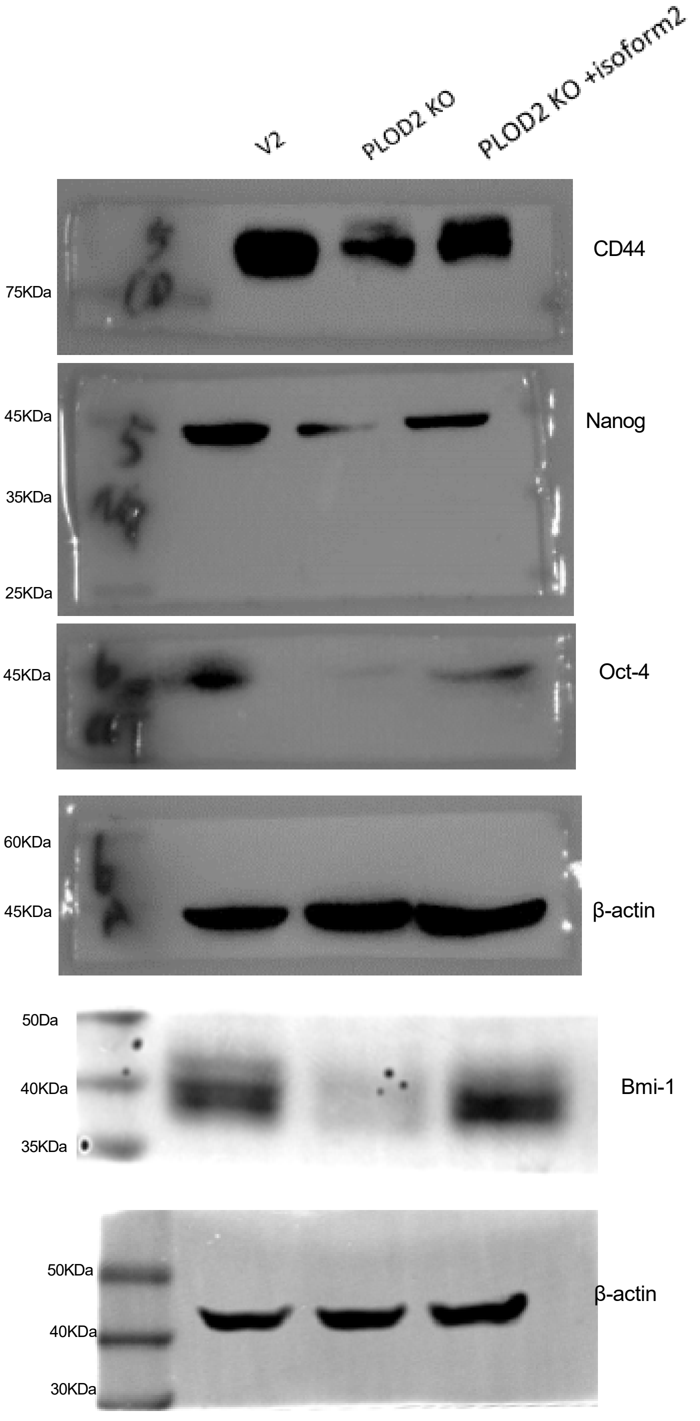

Figure 8A-raw data

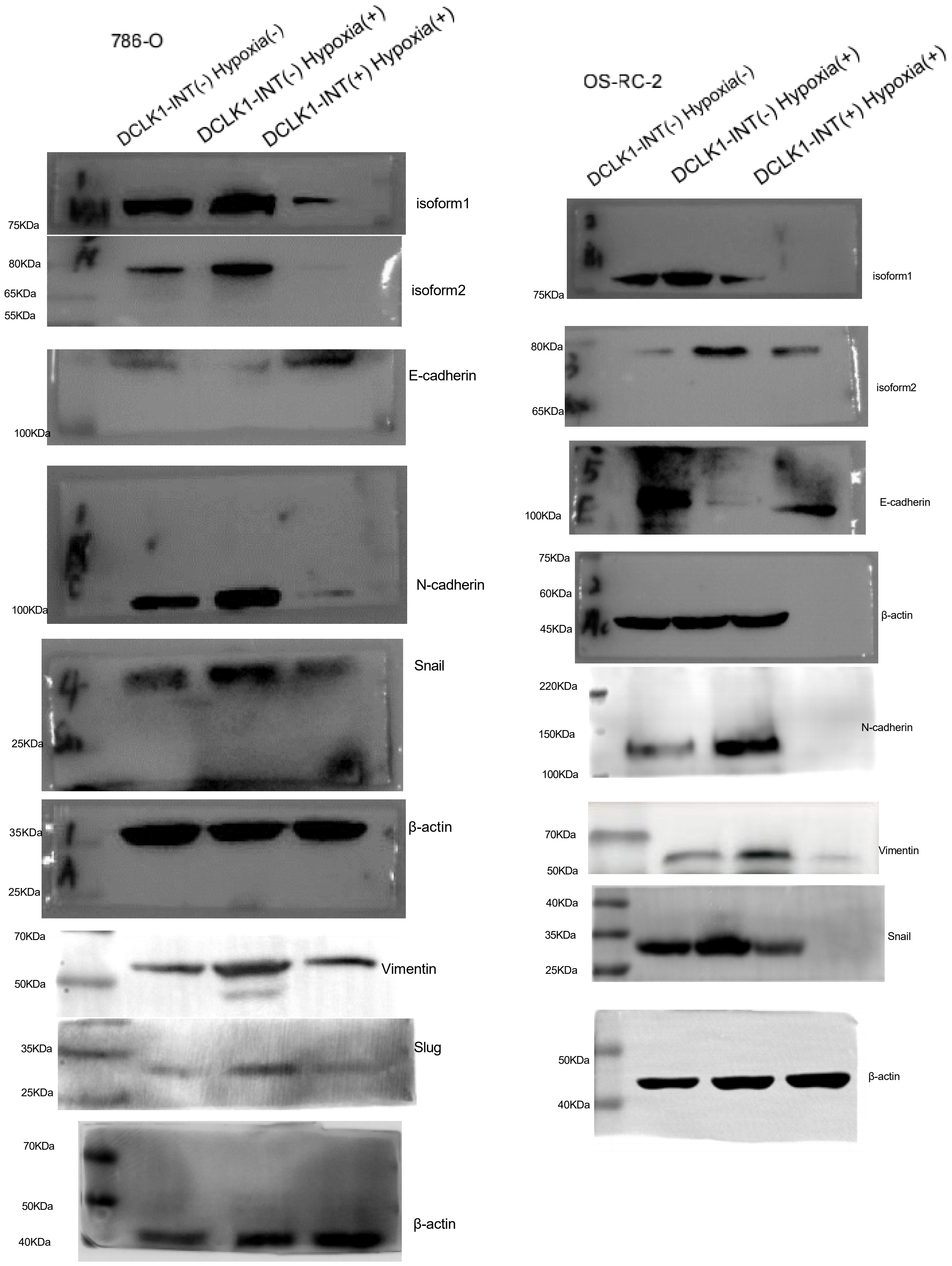

Figure 8B-raw data

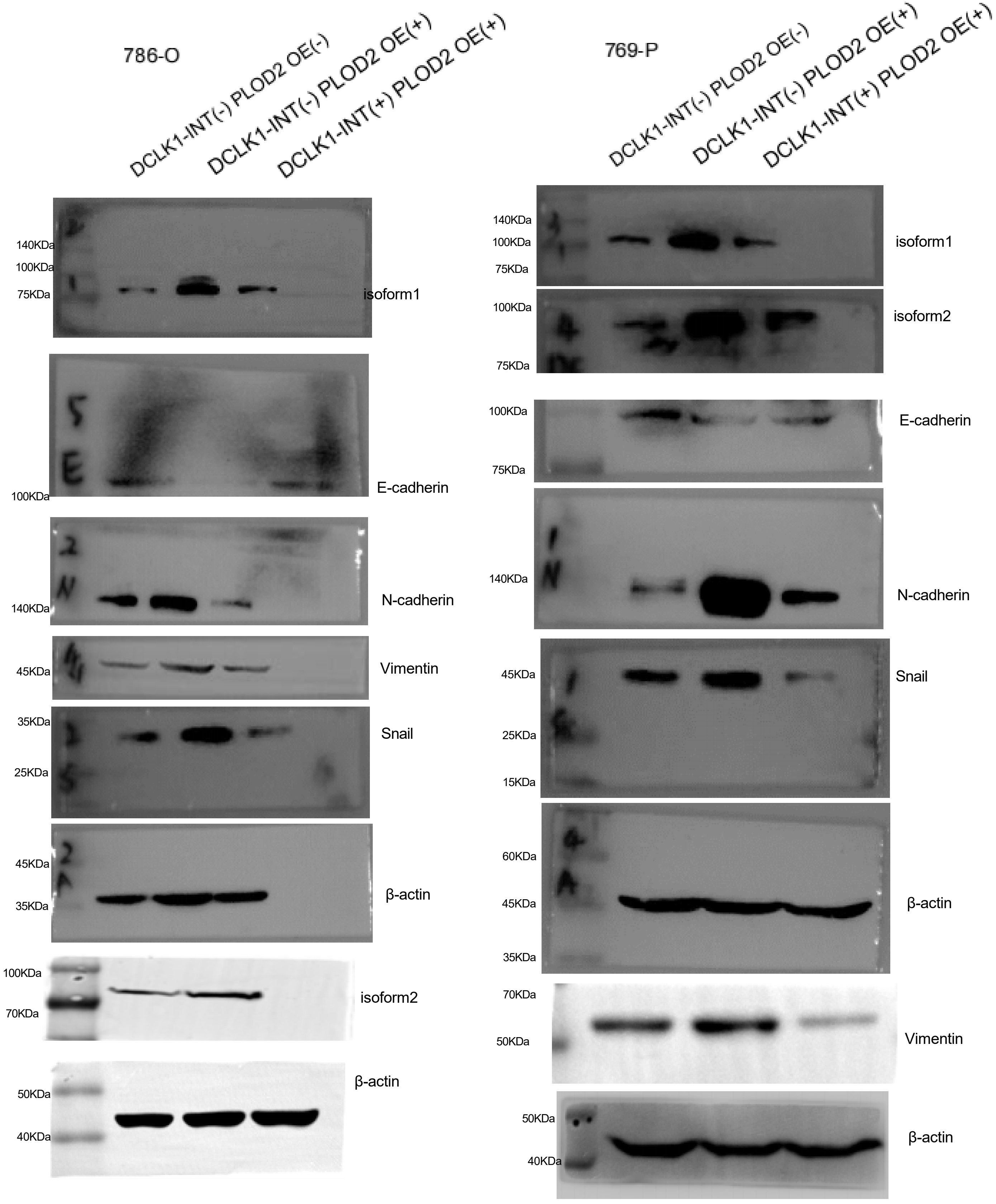

Figure 8E-raw data

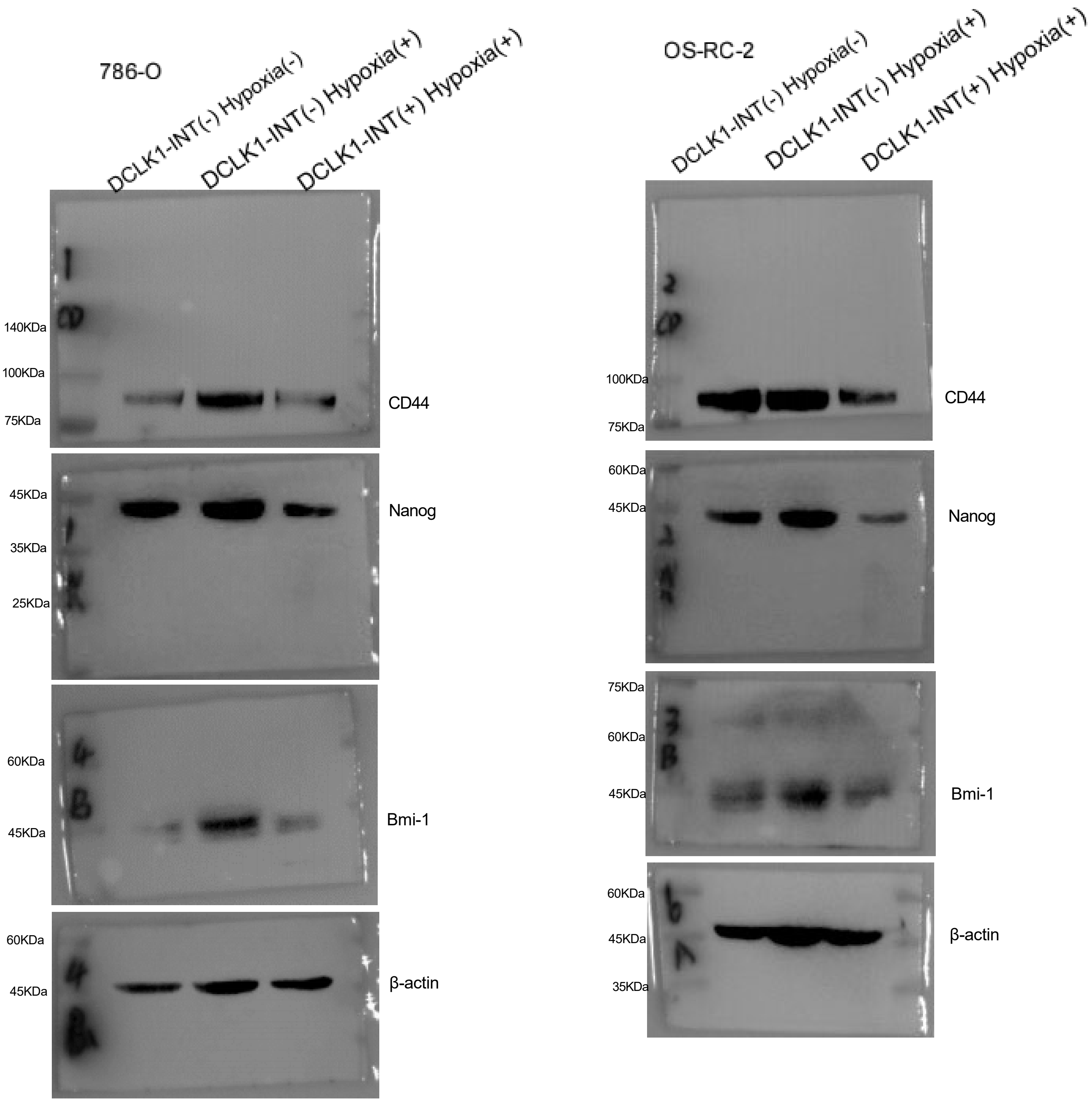

Figure 8G-raw data

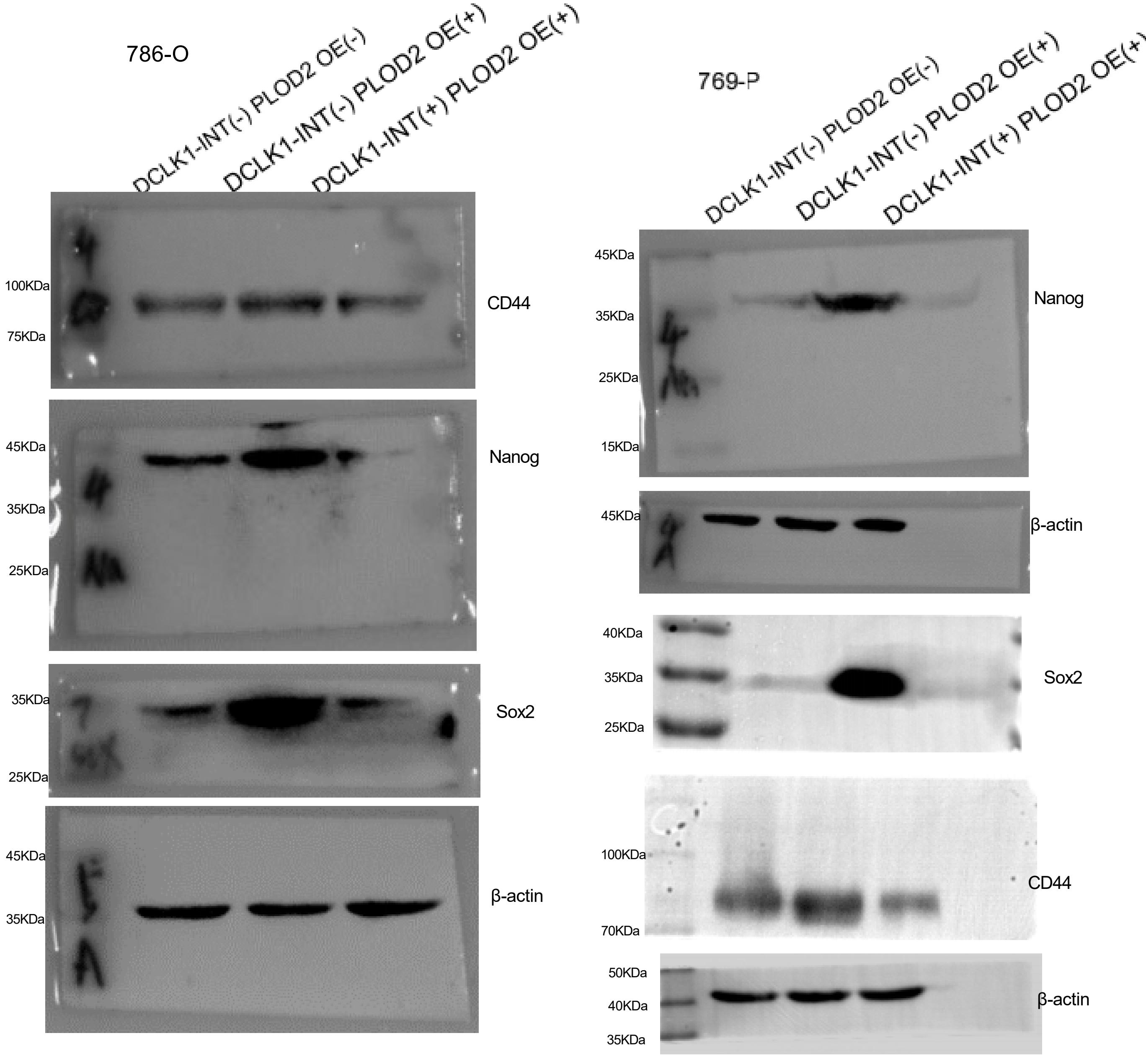

Figure S2-raw data

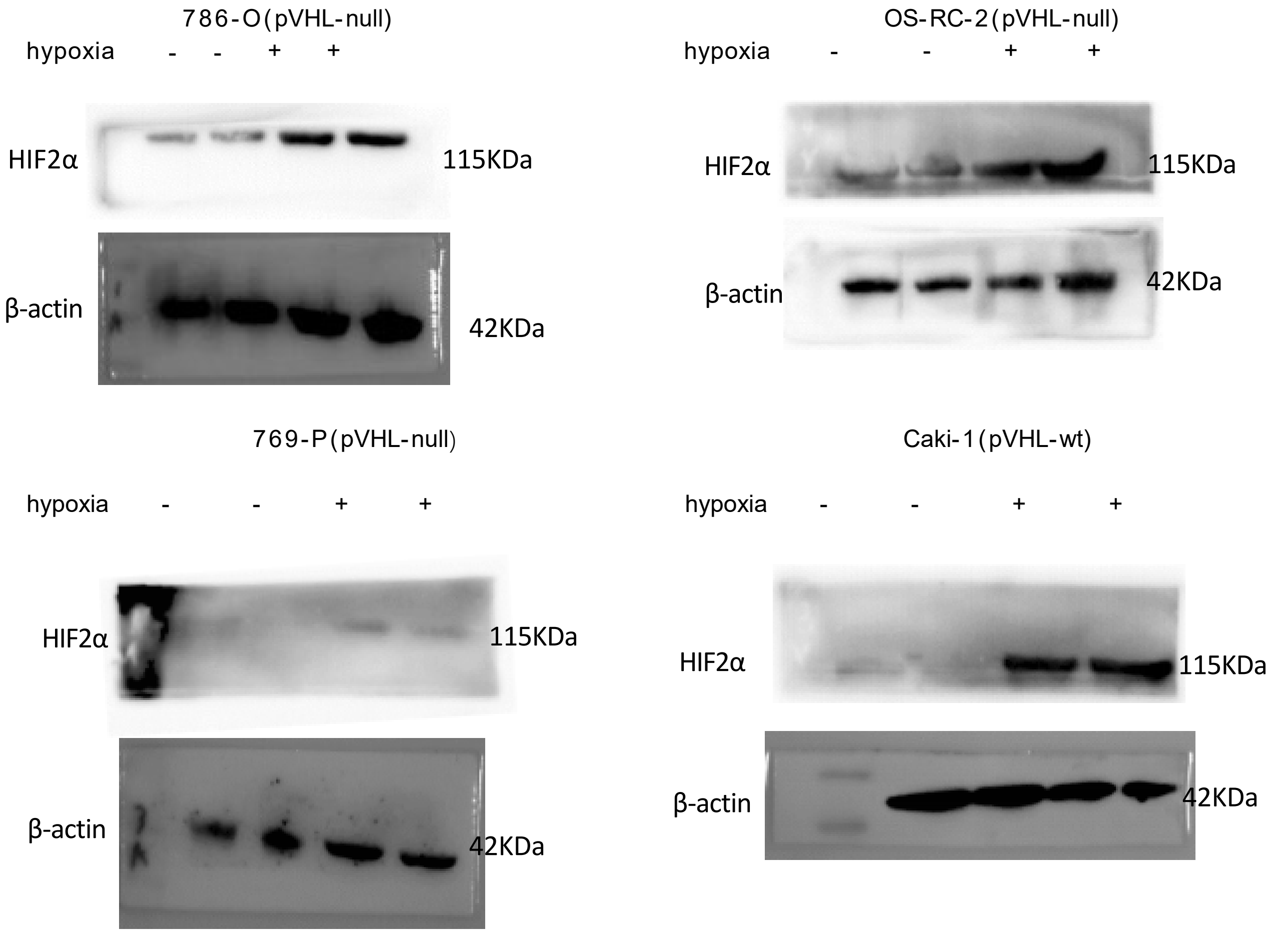

Figure S4-raw data

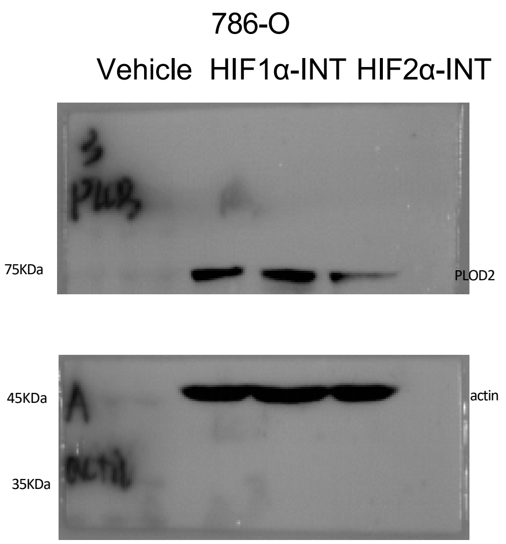

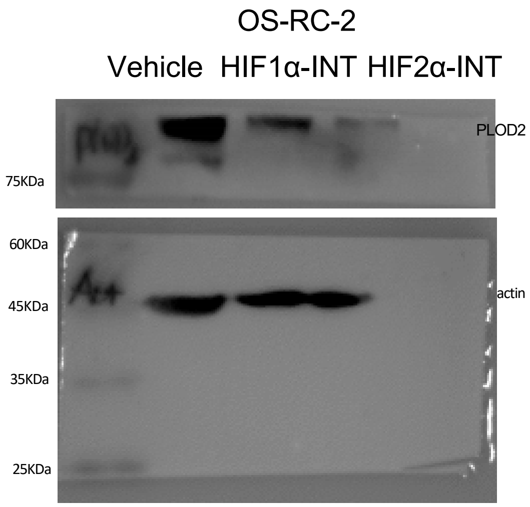

Figure S6A-raw data

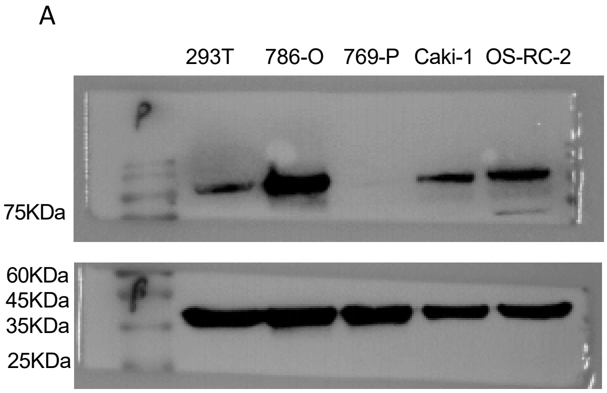

Supplement: Supplementary file 2 — Raw data -Western blot [file 41419_2025_7916_MOESM2_ESM.pdf]
